# Upcycling of waste EPS beads to immobilized codoped TiO_2_ photocatalysts for ciprofloxacin degradation and *E. coli* disinfection under sunlight

**DOI:** 10.1038/s41598-023-41705-1

**Published:** 2023-09-05

**Authors:** Manasa Manjunatha, Hari Mahalingam

**Affiliations:** Department of Chemical Engineering, National Institute of Technology Karnataka (NITK) Surathkal, Mangalore, Karnataka 575025 India

**Keywords:** Catalysis, Photocatalysis, Chemical engineering

## Abstract

The emerging global problem of antimicrobial resistance needs immediate attention. In this regard, this work demonstrates the use of expanded polystyrene waste in the synthesis of immobilized photocatalytic films for the treatment of antibiotics as well as for bacterial disinfection. A boron–cerium codoped TiO_2_ catalyst (of specific composition: B_0.8_Ce_0.2_TiO_2_) was immobilized in an expanded polystyrene (EPS) film prepared from waste EPS beads. These films were studied for the degradation of ciprofloxacin (CIP) and disinfection of *E. coli* under sunlight. The film with a catalyst loading of 20 wt% showed a maximum degradation of 89% in 240 min with a corresponding TOC reduction of 84%. A 7.4 and 6.3 log reduction from the bacterial inactivation studies in the presence and absence of antibiotics, respectively, was obtained. The EPS film was stable after five times of reuse, and no significant chemical changes in the used film were observed from FTIR analysis. The average thickness of the prepared film was found from FESEM analysis to be 1.09 mm. These EPS films were also tested for degradation of other antibiotics, such as norfloxacin, levofloxacin and moxifloxacin. The EPS films were tested in two different reactor volumes at optimum conditions. Also, the effectiveness of B_0.8_Ce_0.2_TiO_2_/EPS film in real water samples indicates its potential in large-scale and real-world applications. Thus, these B_0.8_Ce_0.2_TiO_2_/EPS films can be effectively employed for both degradation of ciprofloxacin and the disinfection of *E. coli* under solar light to solve the increasing problem of antimicrobial resistance.

## Introduction

Only 4% of the total water resources available globally are available as freshwater^1^ to meet the needs of an ever-growing population, and even this abundant water resource is under tremendous strain due to industrialization. Thus, it is essential to find effective methods for the reuse of treated wastewater. In this context, the antibiotic residues present in the water bodies are considered as emerging contaminants due to their role in the development of antimicrobial resistance (AMR)^2^. The problem of AMR is increasing rapidly worldwide. Existing conventional wastewater treatment methods cannot completely remove the antibiotic residues^3^, present in very low concentrations ranging from nano to micrograms per litre. Advanced oxidation processes (AOPs) such as photocatalysis can serve this purpose through the generation of powerful hydroxyl radicals to completely degrade the organic pollutant molecules into less harmful products.

To improve the functionality of titanium dioxide (TiO_2_) because of its wider bandgap of 3.2 eV^4,5^, doping is one strategy to achieve a narrowed bandgap and thus permit utilization of visible light for improved photocatalytic activity^6,7^. Researchers have focused on co-doping with two or more elements, each dopant having a unique property promoting or enhancing the photocatalytic activity under visible light^8–11^. Photocatalysts are commonly used in slurry or suspended forms; however, the photocatalyst is difficult to recover post-treatment and reuse, thus limiting its large-scale applications. These limitations can be overcome by immobilising the photocatalyst onto a suitable inert support. The immobilized form of photocatalyst (nanoparticles) can be recovered and reused easily post-treatment with no or minimal loss of nanoparticles^12,13^.

Immobilized/floating forms of photocatalysts increase the feasibility of catalysts in large-scale applications through their facile recovery and thus reduce operational costs^14–16^. Various supports such as cork^17^, perlite^18,19^, polymers^20–29^, glass beads/glass spheres^30–33^, zeolites^34,35^, clay^36,37^, chitosan^38^, and calcium alginate beads^39,40^ are available for immobilization of the photocatalysts. The supporting material must provide a large surface area, strongly adhere to the catalyst, must have excellent degradation stability against strong oxidative radicals, and must be able to adsorb pollutants onto its surface for efficient photocatalysis.

Polymers are the most suitable among various supports as they are cost-effective and readily available. Polymers include polyethylene, polystyrene, polyamide, polymethyl methacrylate, polyethylene terephthalate, polyvinyl alcohol, polycaprolactone, polyacrylonitrile, polyvinylidene fluoride^23,41,42^. Polystyrene/expanded polystyrene (EPS) beads are widely used as packaging materials (in food, electronic goods, and other fragile products), thus creating an enormous amount of waste polymers that need to be reused in a circular economy. These waste polymers can be upcycled for the development of floating photocatalysts, thus solving a major environmental problem—‘white pollution’^43–45^. The challenge of lesser accessibility to photons by catalysts in the immobilized form can be overcome through the development of floating photocatalysts which use lightweight material as an inert support for immobilization. This floating property also helps in recycling photocatalysts more easily^12,13,46^. The above-mentioned low-density polymers float on the liquid surface (just below the air–liquid interface), thus ensuring effective utilization of light energy for optimum photocatalytic activity^47^.

In recent years, polystyrene/EPS beads in the form of thin films^48–53^ as a support for immobilization have attracted much attention due to their non-toxicity, low cost, and chemical inertness^54^. However, these studies are limited to dyes and disinfection^53,55–57^. Also, these studies have been performed under UV light, as summarized in Table 1 (Supporting Information). Other immobilization studies carried out for the degradation of dyes, antibiotics, and disinfection are summarized in Table 2 (Supporting Information).

So far, very less or no work has been carried out on the degradation of antibiotics and disinfection using boron and cerium codoped photocatalysts and waste polymer support under sunlight. Waste EPS modified into a photocatalytic film was used in the development of a novel reactor for dye wastewater treatment^58^. Also, Varnagaris^59^ explored a modification of waste EPS to an adsorbent material for the removal of norfloxacin antibiotic, while in another study, Liu^57^ demonstrated the use of non-expanded polystyrene beads for bacterial inactivation. Styrofoam-TiO_2_ composite (W-TiEPS)^55^ was studied for methylene blue dye removal and Cr (VI) reduction. This work explores the degradation of ciprofloxacin and microbial disinfection of *E. coli* using an immobilized codoped B_0.8_Ce_0.2_TiO_2_ photocatalyst. The codoped catalyst was synthesized using the facile EDTA-citrate sol–gel method. Here, 0.8 and 0.2 correspond to the atomic percentage of B and Ce dopants, respectively, and this particular catalyst composition was earlier identified as the best-performing photocatalyst in our laboratory^60^. B is a well-known disinfectant and Ce promotes higher adsorption ability^60^. Therefore this synergy of dopants can serve the purpose of efficient degradation and disinfection under sunlight. The immobilized film is made from waste EPS beads which are lightweight and buoyant. Thus, using this film to carry out the degradation of ciprofloxacin (CIP) antibiotic, *E. coli* disinfection studies both in the absence and presence of CIP and its real water applications will help in the development of a sustainable immobilized photocatalyst for large-scale and real-world applications.

## Materials and methods

### Reagents

Degussa P25 Titanium dioxide (99.9% pure) is supplied by Evonik (Japan); boric acid (99.5% extra pure), citric acid (99% extra pure), ammonia solution (99% extra pure), EDTA (99% extra pure) were procured from Loba chemicals (Loba Cheme Pvt Ltd., India); cerium nitrate hexahydrate (99% trace metal basis), acetone (99.5% pure) and ciprofloxacin (≥ 98% HPLC grade) were obtained from Sigma Aldrich Co., USA; L.B. (Luria Bertani) agar and broth were purchased from HiMedia Laboratories Pvt. Ltd., India. Ethanol was procured from Biological Scientific solutions, India. Waste EPS beads were collected from discarded bean bag fillings.

### Preparation of B_0.8_Ce_0.2_TiO_2_/EPS film

**Figure 1:**
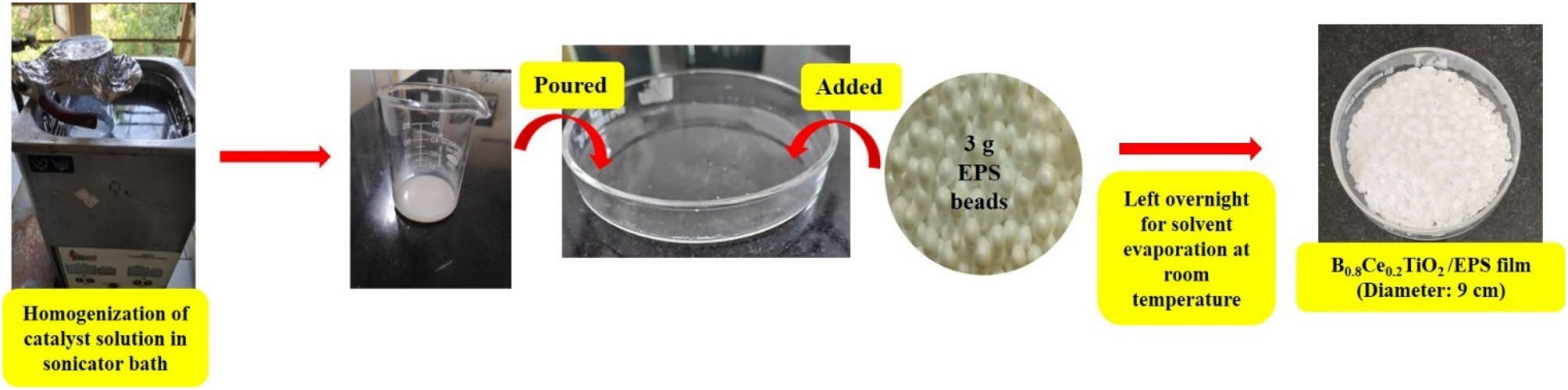
Preparation of B_0.8_Ce_0.2_TiO_2_/EPS film using waste EPS beads and catalyst solution (x wt% w.r.t. weight of EPS beads + 8 mL Acetone + 2 mL Ethanol).

The codoped B_0.8_Ce_0.2_TiO_2_ photocatalyst was synthesized using the facile green EDTA-citrate sol–gel method (see Supporting Information Sect. S2, Scheme 1). The photocatalyst solution (catalyst—a certain weight % with respect to the mass of EPS beads used + 8 mL acetone + 2 mL ethanol) was homogenized through sonication for 15 min in an ultrasonic water bath. The solution was then poured into a Petri plate, followed by the addition of 3 g of EPS (expanded polystyrene) beads. The film was left overnight at room temperature for solvent evaporation (Fig. 1). The prepared film was then gently washed with distilled water to remove loosely bound catalyst particles. The specific amounts of solvents used in the preparation are the optimized amounts based on a preliminary evaluation of the degradation performance for the resulting film (see “Effect of solvent and EPS bead quantities” section).

### Characterization

The B_0.8_Ce_0.2_TiO_2_/EPS film morphology was analyzed using FE-SEM (ZEISS GeminiSEM 300, Oberkochen, Germany) with different magnifications of X100, X500, and X3000. A profilometer measured surface roughness (Taylor Hobson Taly Surf 50, Illinois, U.S.). The XRD analysis (Panalytical Empyrean series 3, Malvern, U.K.) was carried out using Cu Kα (λ = 0.15418 nm) and 2θ range of 5°–80° (with an increment of 0.05°). The composition and chemical states of doped elements were analyzed from XPS (Omicron ESCA+, Chanhassen, U.S.) with an XAl Kα monochromator X-ray source. FTIR-ATR mode (FT-IR Bruker Alpha spectrometer, Bangalore, India) analysis was carried out to determine the functional groups and chemical bonds in the 4000–500 cm^−1^ range A contact angle analyzer (KRŰSS, Drop shape analyzer-DSA 100E, Hamburg, Germany) was used to determine the polarity of the film. The elements leached from the immobilized film were determined using ICP-OES (Agilent Technologies, 5100, California, U.S.). TOC reduction was measured using a TOC analyzer (TOC-V-CSN, Shimadzu, Osaka, Japan). The degraded sample absorbance measurements were recorded using a UV–Vis spectrophotometer (Shimadzu UV-2600, Kyoto, Japan). The degradation products were identified using liquid chromatography-mass spectrometry (Shimadzu LCMS-2020, Kyoto, Japan). 10 µL of the degraded sample was injected and then passed through a 5 µm C-18 column (4.6 × 250 mm, column temperature: 30 °C). At a flow rate of 0.5 mL/min, mobile phase A as 0.1% formic acid in acetonitrile and mobile phase B as 0.1% formic acid in water (16:84) was used. The degraded sample was analyzed for 20 min at a wavelength of 280 nm. The characterization of the catalyst employed in this study (Particle size, DLS, DRS etc.) is given in the earlier study^60^ and the key results from this work are summarized in Supplementary Information S3 Table 3.

### Photocatalytic studies using B_0.8_Ce_0.2_TiO_2_/EPS film

#### Ciprofloxacin (CIP) degradation

Before irradiation, the prepared film was immersed into a 200 mL, 10 ppm ciprofloxacin solution and kept under the dark condition to attain adsorption–desorption equilibrium (30 min with constant stirring). The studies were performed generally for 180 min and in some cases for 240 min (12 to 4 p.m.) under sunlight (at NITK Surathkal, latitude: 13° 0′ 40″ N and longitude: 74° 47′ 44″ E). An average light intensity of 80,000 ± 6000 lx and an average temperature of 32 °C were recorded. Samples were collected at intervals of 15 min, and the absorbance values were analyzed using a UV–Visible spectrophotometer at λ_max_ of 280 nm. The analysis was performed in duplicates and the error bars represent the standard deviation of measurements.

The CIP degradation and mineralization (% degradation and % TOC reduction) was calculated using Eq. (1). Equation (2) gives the pseudo-first-order reaction constant (*k*) as mentioned in the literature.

1 $$\text{\% degradation and \% TOC reduction}=\frac{{C}_{0}-C}{{C}_{0}}\times 100,$$

2 $$-{\text{ln}}\left[\frac{C}{{C}_{0}}\right]=kt,$$

where *C*_0_ &* C*—initial and final CIP concentration/TOC, *t*—reaction time,* k*—rate constant of the reaction.

#### Reusability of B_0.8_Ce_0.2_TiO_2_/EPS film

The stability of the B_0.8_Ce_0.2_TiO_2_/EPS film was determined by reusing the film for five consecutive runs. After each run, the B_0.8_Ce_0.2_TiO_2_/EPS film was gently washed with deionized water and dried overnight at room temperature. For each run (cycle), a freshly prepared CIP solution of 10 ppm was used.

#### Determination of point of zero charge (pzc)

The initial and final pH values were noted for each experiment^61^ which was carried out for different pH values of 3, 5, 7, 9, and 11. The intersection of two plots (initial and final pH) represents the point of zero charge (pzc). The results are plotted in Fig. 11a and it is seen that the B_0.8_Ce_0.2_TiO_2_/EPS film has the point of zero charge at pH 7.64.

#### *E. coli* disinfection (in absence and presence of antibiotic)

Before use, sterilization of glassware was carried out by autoclaving for 20 min at 15 psi. *E. coli* MTCC 9541 strain (isolated from river Ganga) was selected as the model organism for the study and grown using Luria Bertani broth (60 rpm, 24 h). *E. coli* suspension was obtained by centrifuging at 8000 rpm for 10 min (8 °C). Colonies were counted using standard serial dilution and plate count method. A cell density of 75 × 10^8^ CFU (colony forming unit)/mL with a limit of detection of 15 CFU/mL was obtained. The disinfection studies using B_0.8_Ce_0.2_TiO_2_/EPS film were performed in both the absence and presence of antibiotic. The antibiotic resistance assay (see “Determination of point of zero charge (pzc)” section below) was carried out in order to determine the concentration of antibiotic to be used for the disinfection study in the presence of CIP. The batch experiments (200 mL) were carried out for 180 min under sunlight with an initial *E. coli* concentration of 10^8^ CFU/mL. And for experiments in the presence of antibiotic, 1 ppm CIP was used as this concentration did not show any bacterial resistance for *E. coli*. Before irradiation, a dark condition was maintained for 30 min. For every 15 min, samples were collected, serially diluted (as required), plated, and kept for 24 h incubation (37 °C) for colony count. The error bars were obtained for the duplicate measurements.

##### Antibiotic resistance assay

**Figure 2:**
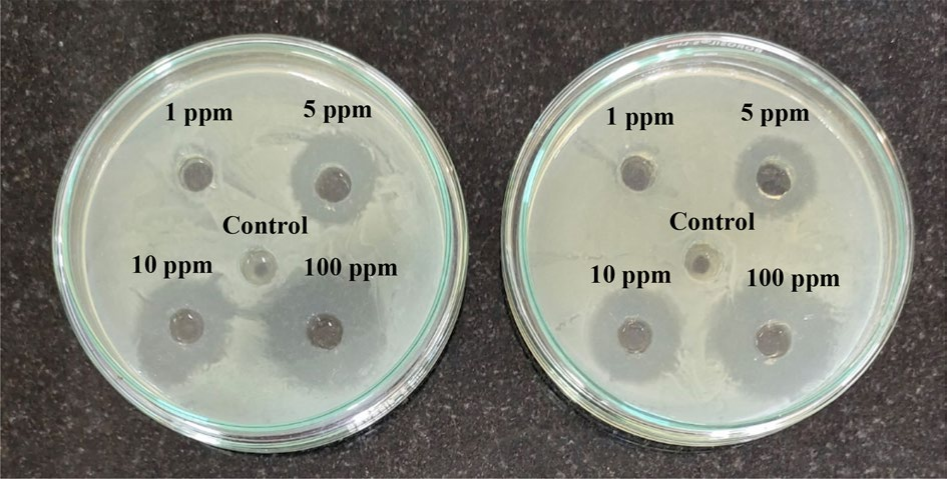
Antibiotic resistance assay using *E. coli* and various concentrations of CIP.

Initially, *E. coli* was tested for its resistance against various concentrations of ciprofloxacin (CIP), namely 1, 5, 10, and 100 ppm, using the spread plate method (Fig. 2). Sterile distilled water was used as the control. Inhibition zones were observed for all concentrations except 1 ppm; thus, resistance was shown by *E. coli* for 1 ppm concentration of CIP. Thus, for the disinfection experiments in the presence of CIP, 1 ppm concentration was used.

## Results and discussion

### Characterization of B_0.8_Ce_0.2_TiO_2_/EPS film

**Figure 3:**
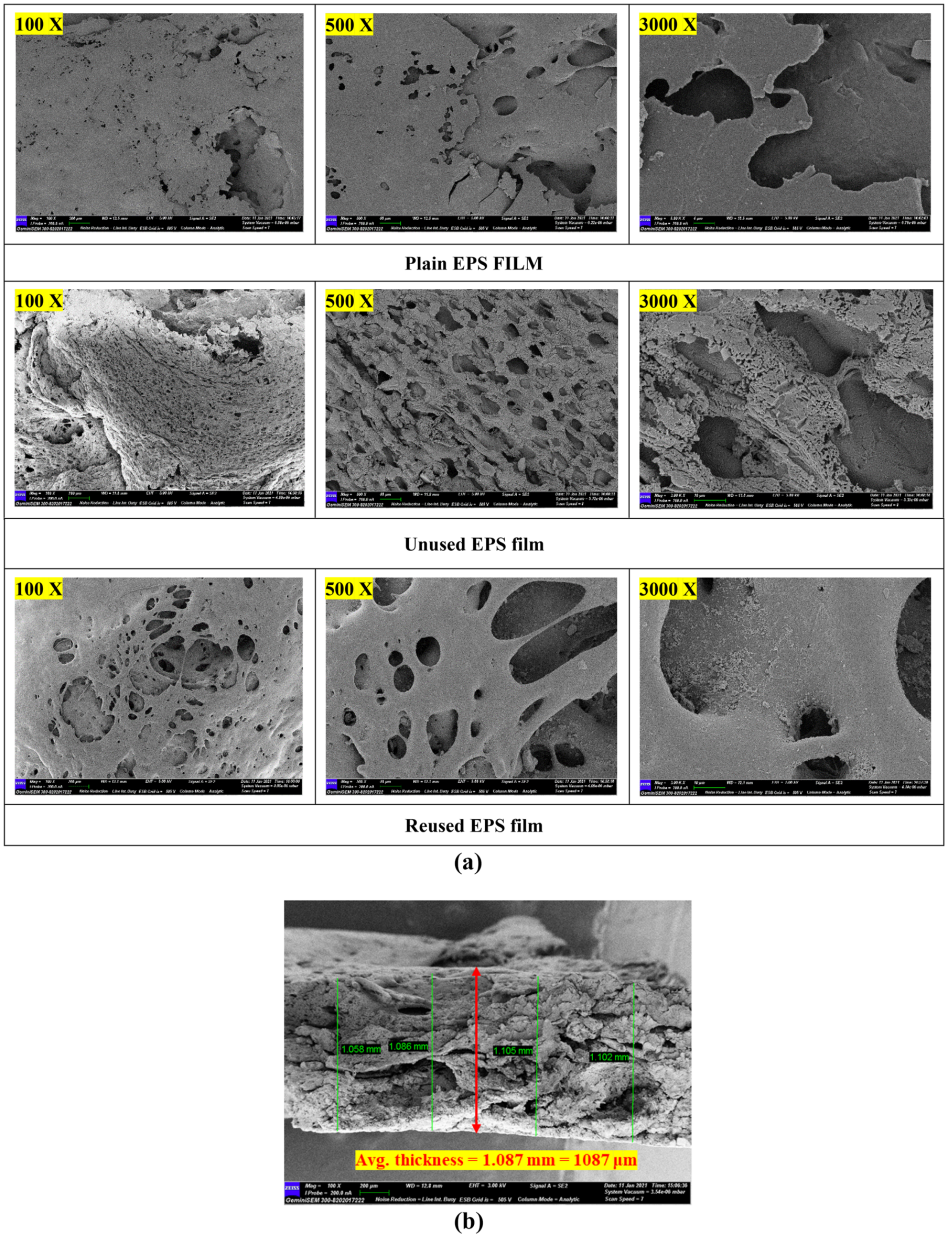
(**a**) FESEM images of EPS film and film with the immobilized catalyst. (**b**) Thickness of the B_0.8_Ce_0.2_TiO_2_/EPS film.

Figure 3a shows the morphology of plain EPS film, unused EPS film with B_0.8_Ce_0.2_TiO_2_ catalyst particles distributed on the surface of the film. Pores created on the surface of the EPS film are clearly visible, and these pores act as active sites for photocatalytic activity. After reusing the EPS film five times, no significant morphological changes were observed. From the FESEM analysis, an average thickness of 1087 μm (Fig. 3b) and an average roughness of 15.4323 μm was observed from profilometer measurements.

**Figure 4:**
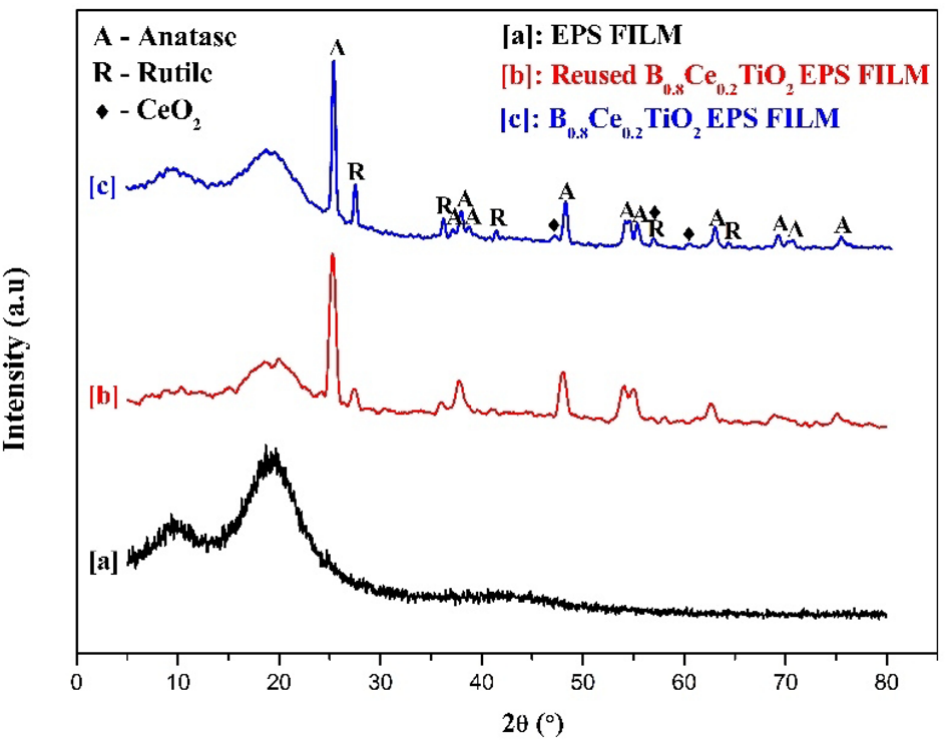
XRD spectra of (a) EPS film, (b) B_0.8_Ce_0.2_TiO_2_/EPS film after using five times, (c) B_0.8_Ce_0.2_TiO_2_/EPS film.

The XRD spectra of plain EPS film, B_0.8_Ce_0.2_TiO_2_/EPS film, before and after five times of reuse are given in Fig. 4. In the plain film, two peaks were observed at 9.975°—amorphous polymer and 19.625°—crystalline polymer^62^. After the catalyst immobilization, crystalline peaks of TiO_2_ (anatase and rutile) and cerium peaks were observed (TiO_2_—ICSD no. 9852 (Anatase), 9161 (Rutile) and CeO_2_—ICSD no. 072155). The peaks at 25.32° (101), 37.01° (103), 37.87° (004), 38.60° (112), 48.09° (200), 54.32° (105), 55.12° (211), 62.73° (204), 68.97° (116), 70.30° (220), and 75.11° (215) correspond to anatase TiO_2_^63–65^. The peaks at 27.46° (110), 36.11° (101), 41.31° (111), 56.72° (220), and 64.04° (310) correspond to rutile TiO_2_^65,66^. Cerium peaks were found at 47.05° (220), 56.72° (311), and 60.22° (222)^67–69^. No changes in the XRD pattern were observed after five times of reuse of the film. This indicates that the degree of crystallinity remains unchanged even after exposing the film to sunlight.

**Figure 5:**
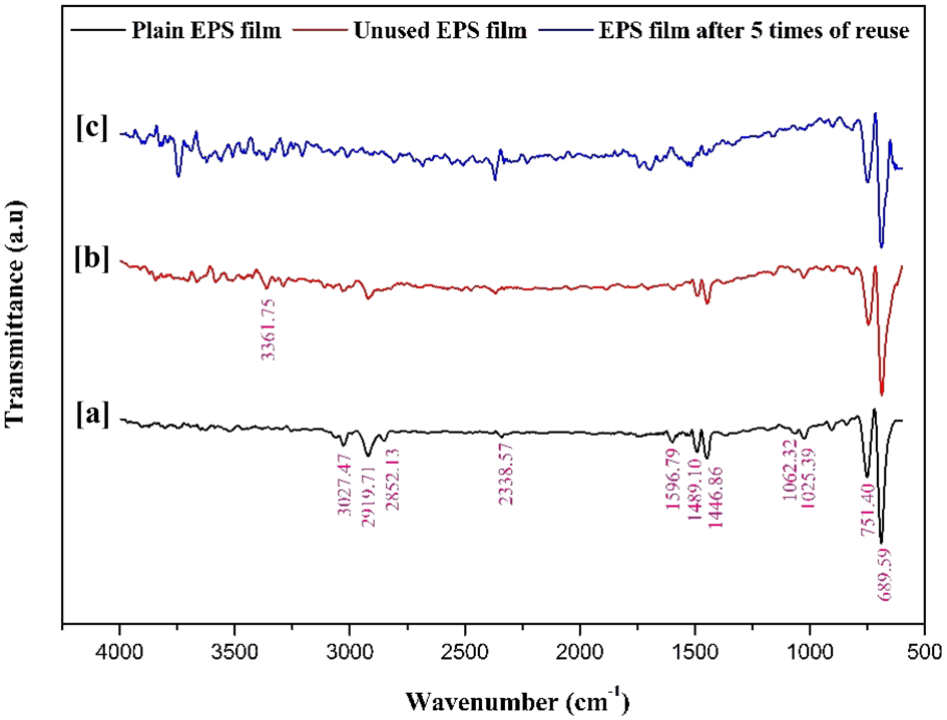
FTIR spectra of (a) plain EPS film, (b) unused EPS film, and (c) EPS film after five times of reuse.

Figure 5 shows the FTIR spectra of the plain EPS film, unused film, and film after five times of reuse. The peak at 3361.75 cm^−1^ corresponds to the O–H stretch. The next peak observed at 3027.47 cm^−1^ corresponds to the C–H bond of the benzene ring in polystyrene. The third peak at 2919.71 cm^−1^ is due to C–H stretching vibration. Peaks between 1597 and 1446 cm^−1^ correspond to aromatic C=C bond stretch. The next two peaks at 751.40 cm^−1^ and 689.59 cm^−1^ correspond to the aromatic C–H bend. No major chemical changes were observed after five times of reuse of the film. No additional peaks were observed, which suggests the absence of contaminants. Similar results were observed in the literature^70,71^.

From contact angle measurements, 82.93° was measured for the B_0.8_Ce_0.2_TiO_2_/EPS film, which indicates that it is hydrophilic. TiO_2_ is hydrophilic; B_0.8_Ce_0.2_TiO_2_ photocatalyst and EPS beads are hydrophobic. After catalyst immobilization, it is seen that the EPS film behaves as hydrophilic.

**Figure 6:**
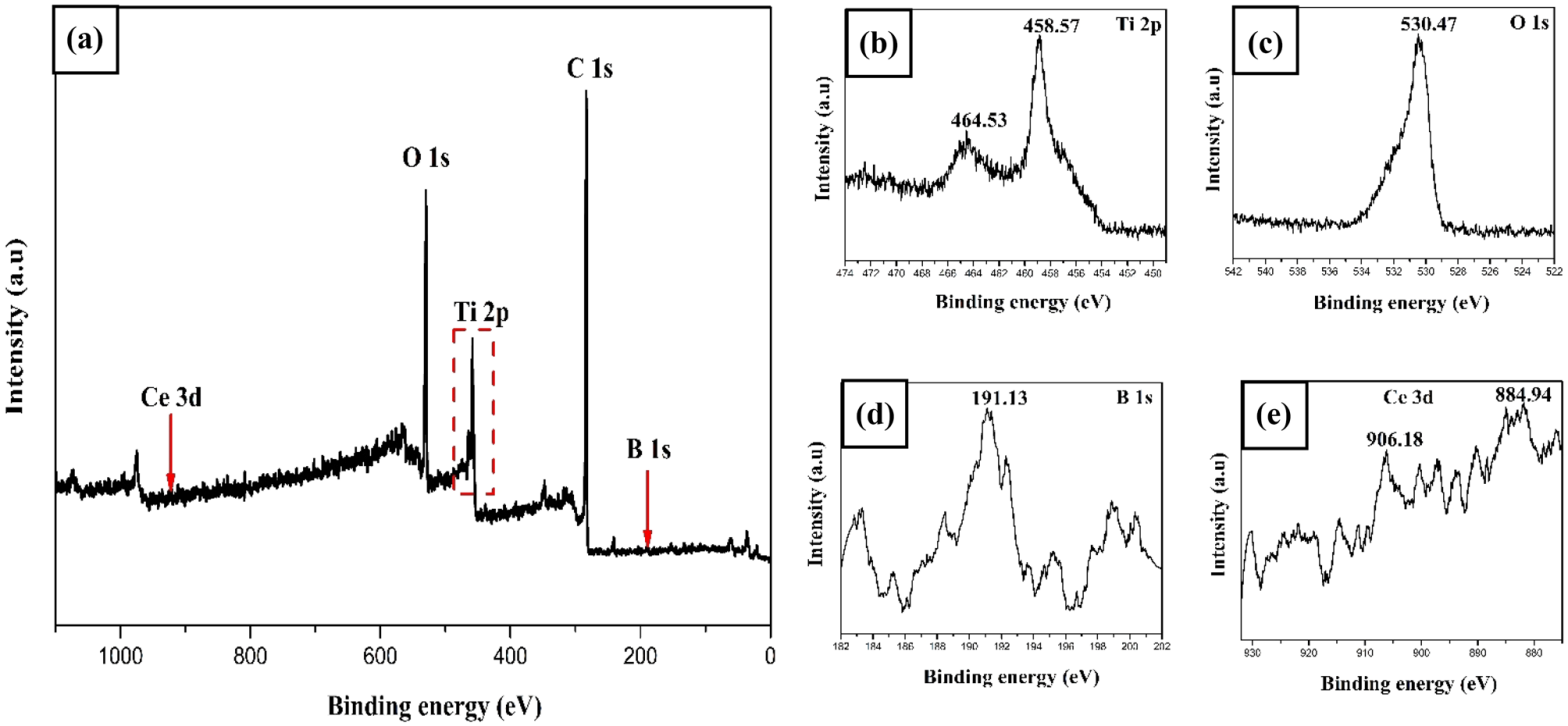
(**a**) XPS analysis of the immobilized B_0.8_Ce_0.2_TiO_2_ photocatalyst film. **(b)** Ti spectra, **(c)** O spectra, **(d)** B spectra, **(e)** Ce spectra.

The composition and chemical states of the B_0.8_Ce_0.2_TiO_2_ immobilized photocatalyst were determined from XPS analysis. Figure 6a depicts the survey spectra of B_0.8_Ce_0.2_TiO_2_ immobilized film. The Ti 2p peaks were observed at 458.57 eV (corresponds to 2p_3/2_ orbit) and 464.53 eV (corresponds to 2p_1/2_ orbit) (Fig. 6b) which corresponds to Ti^4+^^10^. One O 1s peak was observed at 530.47 eV (Fig. 6c), which corresponds to the surface OH^−^ group/lattice oxygen^8,64,72^. A peak at 191.13 eV (Fig. 6d) for B 1s indicates the interstitial lattice position of B, which promotes the reduction of Ti^4+^ to Ti^3+^ and Ti^3+^ facilitates effective separation of photogenerated charges and thus enhances the photocatalytic activity^8,10,73,74^. Two Ce 3d peaks (Fig. 6e) appeared at 884.94 eV and 906.18 eV. This indicates the presence of Ce^4+^^10,75^. From the XPS study, the amount of photocatalyst present in the EPS film was quantitatively analyzed (Table 4 of SI), and the actual amount of B_0.8_Ce_0.2_TiO_2_ photocatalyst present was 19.96 wt%, which is in agreement with the intended catalyst loading (20 wt%).

Leaching of the photocatalyst from the EPS film was studied by subjecting the film to continuous stirring in 200 mL distilled water for 24 h. ICP-OES analysis was used to quantify the concentration of elements leached from the photocatalyst. The results showed minute traces of boron (0.3 ppm) and cerium (0.09 ppm). The Boron concentration values are within the permissible limits specified in the drinking water standards set by WHO (2008)^76,77^. No traces of elemental Ti were observed.

### Optimization of CIP degradation parameters

#### Effect of solvent and EPS bead quantities

**Figure 7:**
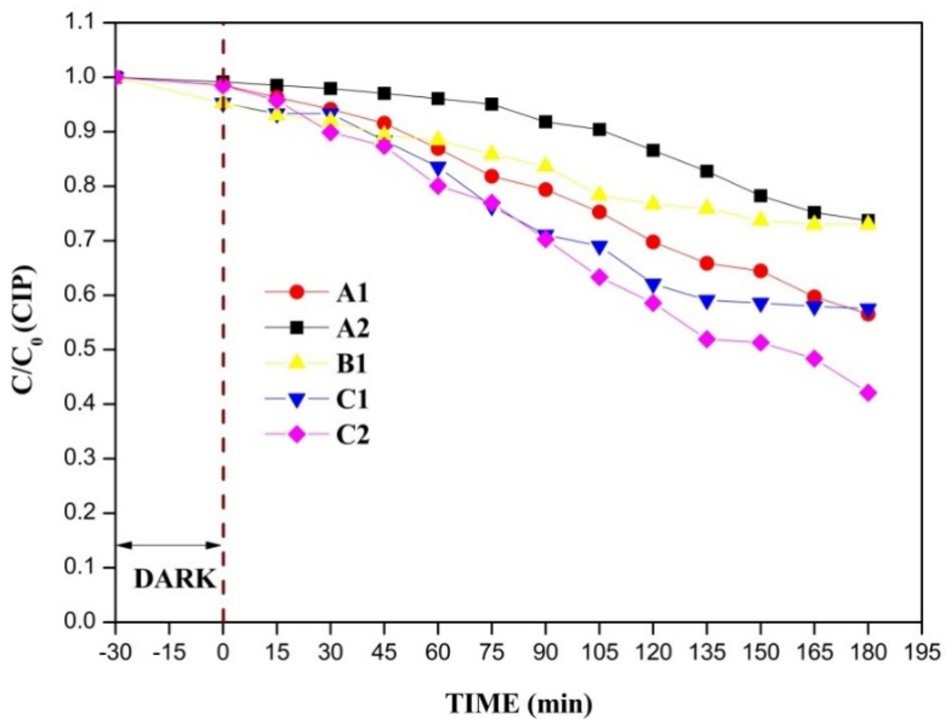
Effect of solvents on the photocatalytic degradation of CIP using B_0.8_Ce_0.2_TiO_2_/EPS film (A1: 3 g EPS + 10 mL Acetone (5 wt%), A2: 3 g EPS + 10 mL Acetone (10 wt%), B1: 4 g EPS + 10 mL Acetone (5 wt%), C1: 3 g EPS + 8 mL Acetone + 2 mL Ethanol (5 wt%), C2: 3 g EPS + 8 mL Acetone + 2 mL Ethanol (10 wt%)).

**Figure 8:**
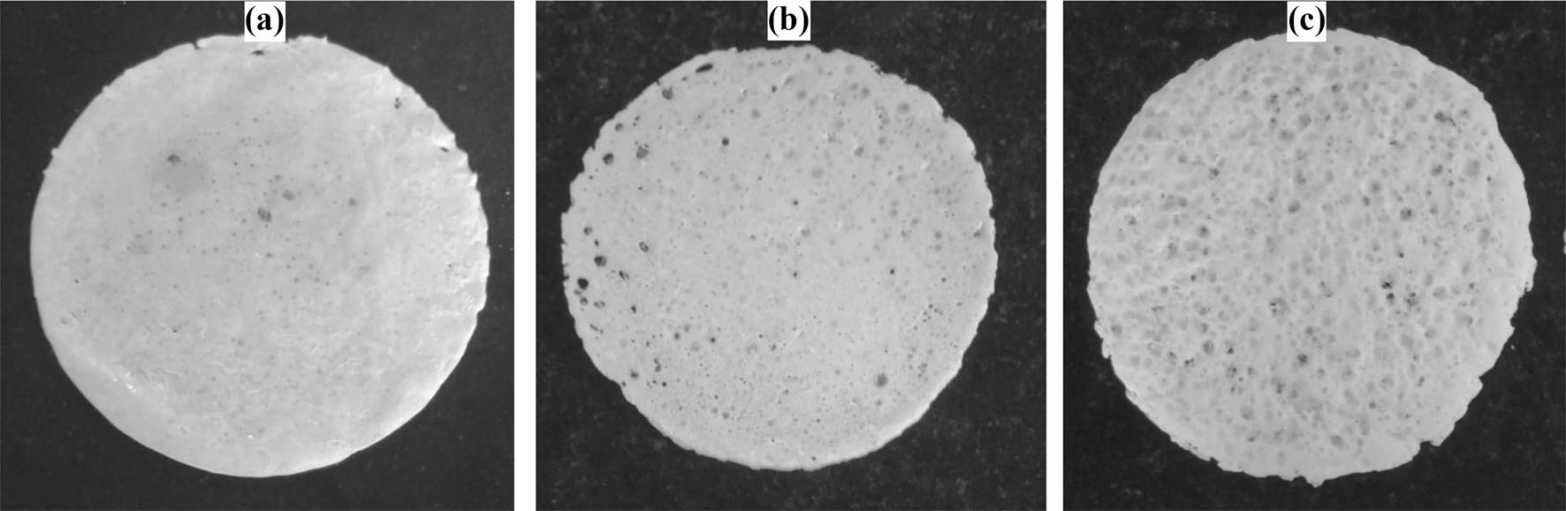
EPS films prepared by varying the quantity of EPS and solvents: (**a**) 3 g EPS + 10 mL Acetone, (**b**) 4 g EPS + 10 mL Acetone, and (**c**) 3 g EPS + 8 mL Acetone + 2 mL ethanol.

The solvents i.e., acetone and ethanol, were combined in different ratios (10 mL acetone and 8 mL acetone + 2 mL ethanol), and the amount of EPS beads was varied (3 g and 4 g) to check the resulting performance of the EPS film with respect to the degradation of CIP. The degradation efficiency was found to be only 26% for 5 wt% (A1 in Fig. 7) and 43% for 10 wt% (A2 in Fig. 7) of B_0.8_Ce_0.2_TiO_2_/EPS film prepared with 10 mL acetone and 3 g EPS. With the addition of 8 mL acetone and 2 mL ethanol, the degradation efficiency was found to be increasing, with 42% degradation observed for 5 wt% (C1 in Fig. 7) and 57% for 10 wt% (C2 in Fig. 7). Only 27% degradation was observed for the film with 4 g EPS and 10 mL acetone (B1 in Fig. 7). As mentioned in the literature^78^, the degradation increases with an increase in the polarity of the solvent, which is observed in this study after the addition of ethanol. Also, acetone is considered to be less toxic than the other industrial solvents^79^. The presence of pores (as observed from Fig. 8) indicates the existence of active sites, which helps in achieving higher photocatalytic activity.

#### Effect of catalyst dosage

**Figure 9:**
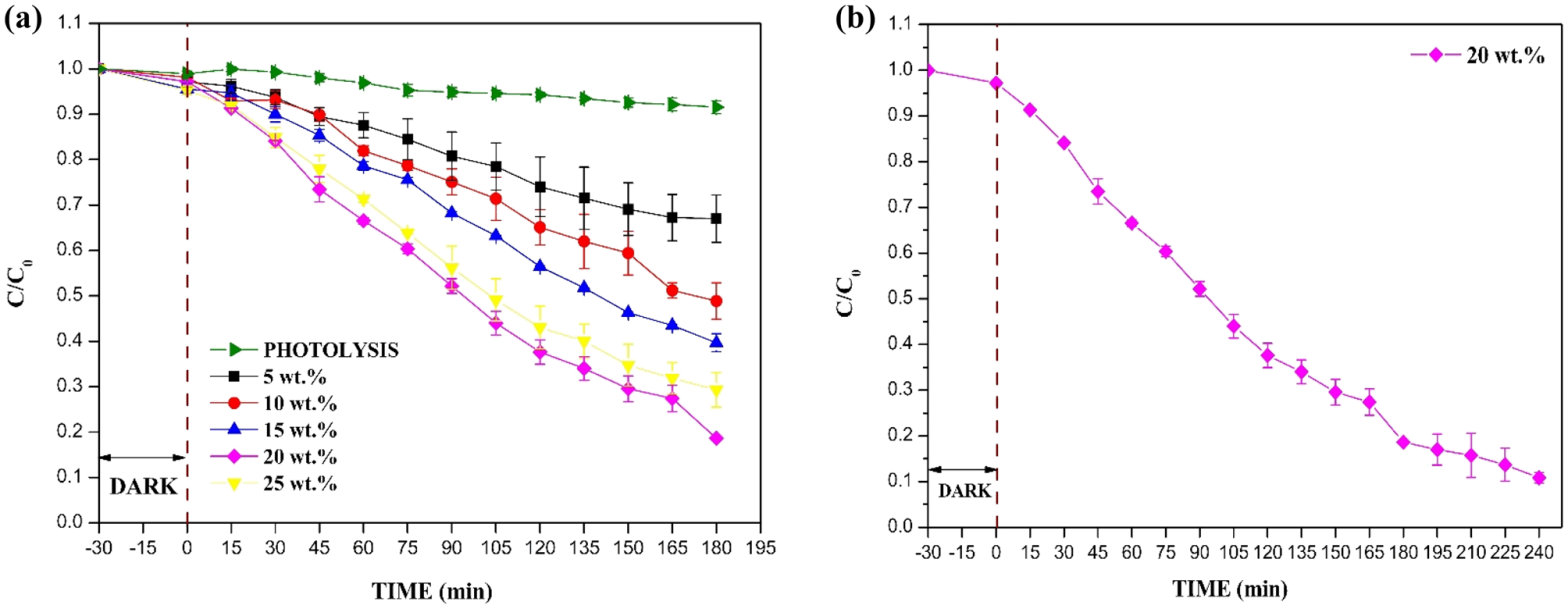
(**a**) Effect of catalyst dosage in immobilized EPS film. (**b**) Photocatalytic degradation of CIP for 20 wt% EPS film for longer time period. Experimental condition: Initial concentration of CIP = 10 ppm, under sunlight.

Photolysis experiments showed only 8.44% degradation of CIP under sunlight, thus indicating the need for further treatment using the B_0.8_Ce_0.2_TiO_2_/EPS film. The effect of catalyst dosage on photocatalytic degradation of CIP was examined by varying the catalyst dosage as 5, 10, 15, 20, and 25 wt% (Fig. 9a). A maximum degradation of 81.36% was observed for 20 wt% after 180 min under sunlight. The degradation efficiency of CIP was found to be increasing with an increase in the catalyst dosage up to 20 wt% (optimum catalyst dosage). An increase in the catalyst dosage results in an increase of electron–hole pairs, which in turn improves photocatalytic degradation. However, beyond the optimum catalyst dosage, the degradation decreased due to the reduction in pore size and the number of pores due to the agglomeration of the higher number/excess catalyst particles on the film’s surface. Similarly, in the literature^71^, photocatalytic activity has decreased with an increase in the catalyst dosage. Further, for the 20 wt% photocatalytic film, a final degradation of 89.17% (Fig. 9b) was observed at the end of 240 min.

#### Effect of CIP initial concentration and kinetic studies

**Figure 10:**
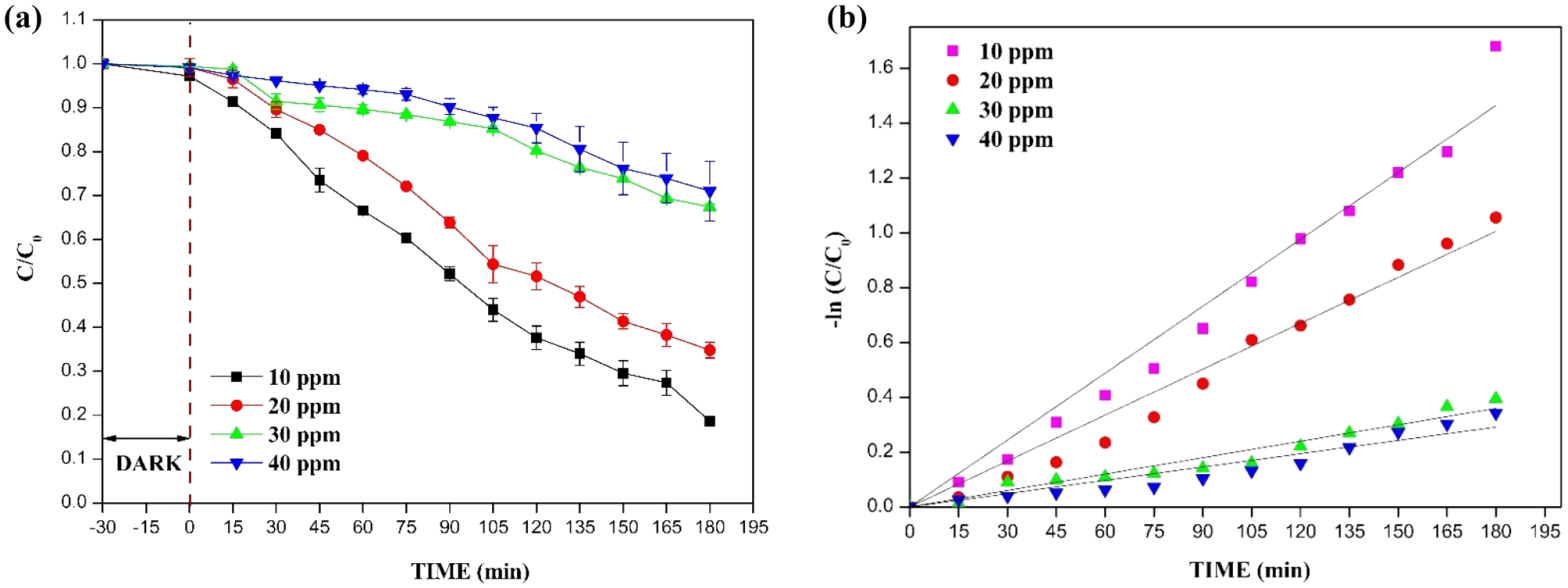
(**a**) Effect of CIP initial concentrations for 20 wt% EPS film, (**b**) Kinetics plot.

The effect of CIP initial concentration on the degradation was studied with the optimum catalyst dosage of 20 wt% by varying the initial concentration of CIP in the range of 10 to 40 mg/L, as shown in Fig. 10a. The degradation was found to be decreasing with an increase in antibiotic concentration. With an increase in the CIP concentration, the reactive species (^·^OH radicals) remain constant for a given catalyst dosage and time of irradiation. A higher pollutant concentration requires a higher number of radicals for degradation. Also, more pollutant molecules compete for the same active sites in catalysts, thus decreasing degradation efficiency. Similarly, in the literature^71^, there is a decrease in the degradation with an increase in the initial concentration of CIP.

The reaction kinetics of CIP degradation for the above different initial concentrations is shown in Fig. 10b. The kinetics of the reaction was found to be pseudo-first-order. The *k* and R^2^ values are given in Table 5 (Supporting Information—Sect. S4). The rate constant *k* values were found to be decreasing with an increase in the initial concentrations of CIP.

### Effect of pH

**Figure 11:**
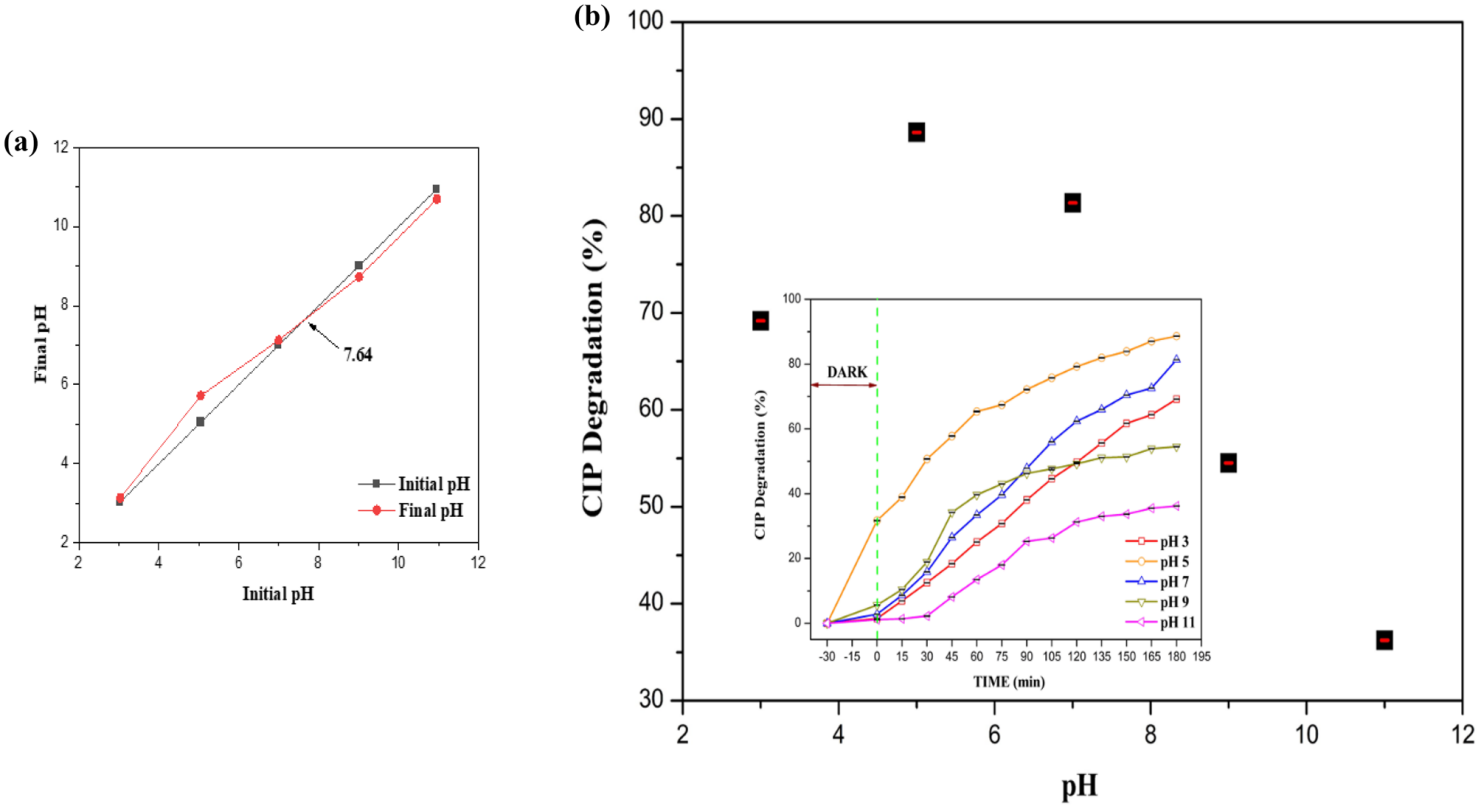
(**a**) Point of zero charge (pzc) of B_0.8_Ce_0.2_TiO_2_/EPS film and **(b)** effect of pH on CIP degradation.

The pH of a solution is an important aspect as it determines the properties of the surface charge of the photocatalyst and the adsorption behaviour of pollutant molecules^80,81^. It can affect changes in the ionic state of pollutants, the surface charge of the photocatalyst, and concentrations of reactive oxygen species. Thus, pH greatly influences photocatalytic degradation of the CIP^82–84^. The effect of pH on CIP degradation was studied by varying the pH as 3, 5, 7, 9, and 11 using the optimum conditions of 20 wt% catalyst-loaded film and 10 ppm of CIP for 180 min. pH was adjusted using 1 M HCl and 1 M NaOH. The CIP has two dissociation constant values of pKa_1_ = 6.09 (carboxylic acid group) and pKa_2_ = 8.74 (nitrogen on piperazinyl ring). The point of zero charge of B_0.8_Ce_0.2_TiO_2_ was found to be 7.64 (Fig. 11a). The CIP degradation was good at both pH 7 and pH 5. From Fig. 11b (the inset figure shows the degradation for different pH values over a period of 180 min), at high pH values (basic pH), both the EPS film and CIP are negatively charged, due to which repulsion occurs, and thus, a lesser degradation happens. The EPS film and CIP are positively charged at low pH values (acidic pH). However, degradation is higher than that at the high pH, which might be due to the higher oxidation potential of hydroxyl radicals for the degradation of organic pollutants at low pH^84^. The pH of rivers and lakes in India lies in the range of 6.5–8.5. Also, the effluent with antibiotic residues from the pharmaceutical manufacturing industries or hospital effluent has a pH in the range of 6.7–7.7 (effluent discharge standards for industries and hospitals)^80^. In view of practical and real water sample applications, all the studies were performed at a pH of 7.

### Reusability studies

Reusability studies of the B_0.8_Ce_0.2_TiO_2_/EPS film were performed five times with a degradation time of 180 min for each cycle. After each cycle, the EPS film was washed with distilled water and dried at room temperature (overnight). The degradation efficiency of CIP slightly decreased from 81.36 to 71.15% (Fig. 2, Supporting Information Sect. S5) after five consecutive cycles of reuse, and this decrease is lesser when compared to the suspended form with a ~ 20% decrease in the degradation efficiency after five times of reuse of the nanoparticles in the earlier study^60^. The slight decrease in degradation efficiency can be attributed to the adsorbed CIP on the surface of the film, which blocks the active sites available for photocatalytic activity. Thus, these results suggest the stability of the B_0.8_Ce_0.2_TiO_2_/EPS film and its application for large-scale purposes.

### Analysis of degraded CIP samples

#### TOC analysis and antimicrobial activity of the degraded CIP sample

The degraded CIP sample showed a TOC reduction of 46.41% after 180 min and 84.41% after 240 min. These results indicate the mineralization of CIP with an increase in the treatment time. Further, the loss of antimicrobial activity of the degraded CIP sample was determined using *E. coli* as the test organism by the agar well diffusion method. The experimental procedure is given in the Supporting Information Sect. S8. As seen from Fig. 3 (Supporting Information—Sect. S6), the zone of inhibition was found to be decreasing with an increase in the treatment time, with no inhibition zones being observed after 195 min, and this is in accordance with the degradation observed (Fig. 2). The decrease in the zone of inhibition suggests the loss of antibiotic activity in the degraded sample.

#### LC–MS analysis

**Figure 12:**
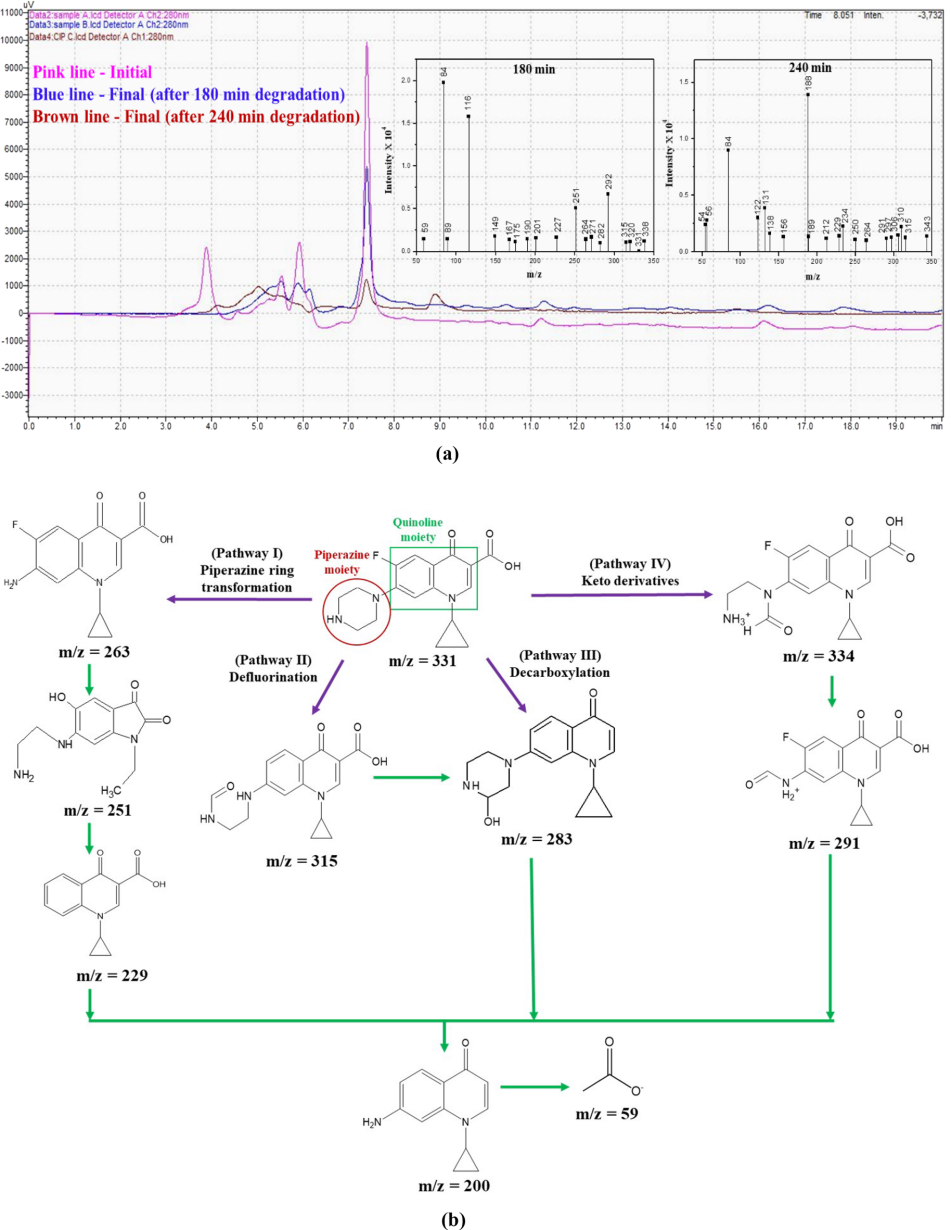
(**a**) HPLC and MS spectrum of CIP degraded products using B_0.8_Ce_0.2_TiO_2_/EPS film. **(b)** Possible degradation pathways of CIP from LC–MS analysis.

Figure 12a illustrates the HPLC peaks of the initial (pink line) and final (blue line—after 180 min, brown line—after 240 min) CIP samples. The inset graph depicts the MS spectrum of the degraded products. The obtained (experimental) m/z values were compared with m/z values in the literature, and the degraded product structures were elucidated (Table 6, Supporting Information Sect. S7).

The possible pathways of CIP degradation using B_0.8_Ce_0.2_TiO_2_/EPS film under sunlight are given below in Fig. 12b. The degradation has occurred mainly through the transformation of the piperazine ring, defluorination, decarboxylation, formation of keto derivatives, and finally, less harmful & low molecular weight products were formed. In pathway I, the degradation has occurred via the transformation of the piperazine ring with the formation of m/z: 263 (degradation of piperazine moiety through the loss of CO_2_ from the carboxylate group) → 251 (piperazine ring cleavage through loss of –C_2_H_2_) → 229 (elimination of piperazine moiety). In pathway II, there is formation of the product with m/z: 315 → 283, through the loss of F atom (defluorination). In pathway III, the m/z: 283 was formed by the removal of carboxyl group (decarboxylation). In pathway IV, the formation of keto derivatives has occurred corresponding to the m/z: 334 → 291. Finally, the product with m/z: 200 was formed. Finally, low molecular weight (m/z: 59) compounds and less harmful products were formed.

### Disinfection studies of the B_0.8_Ce_0.2_TiO_2_/EPS film and application in real water matrices

**Figure 13:**
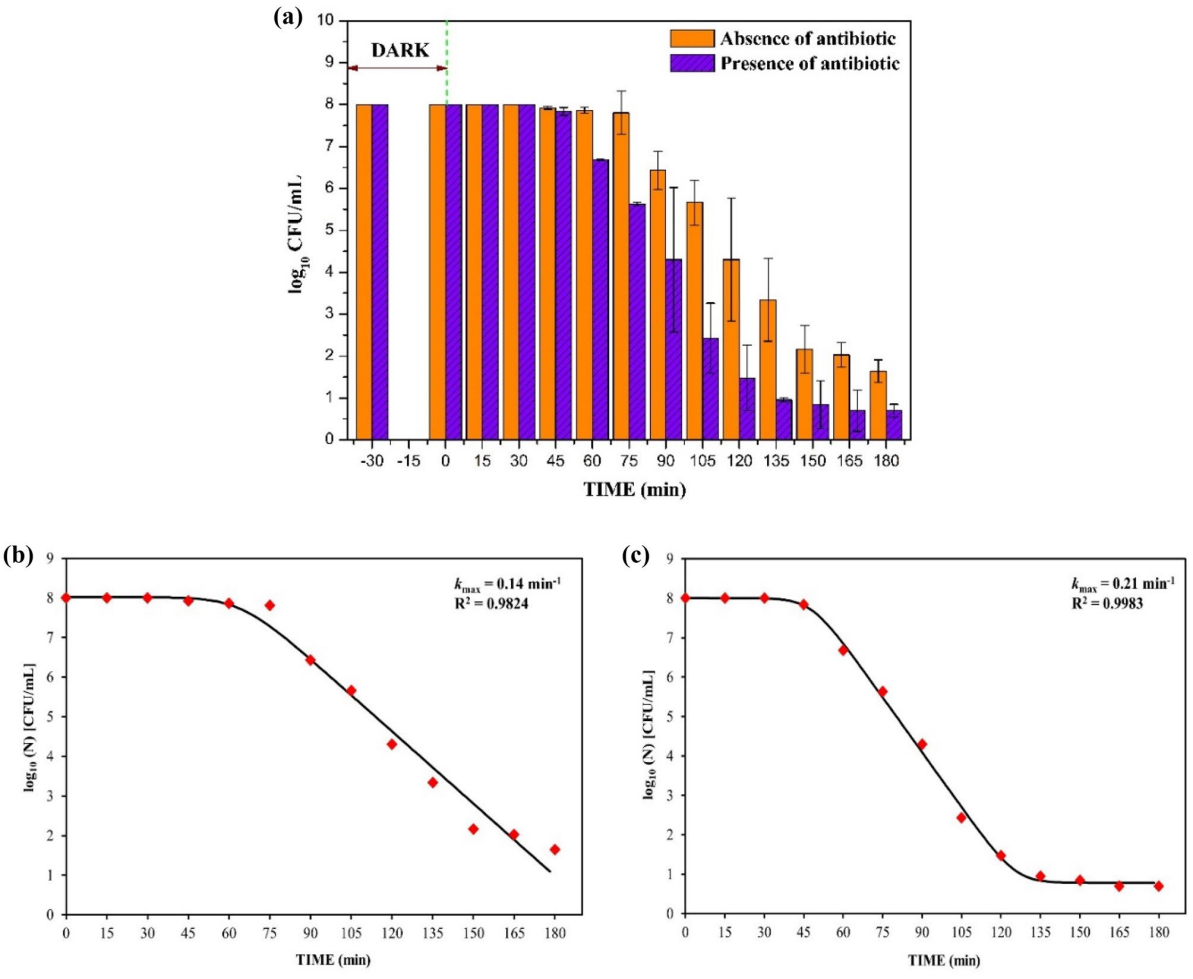
**(a)** Disinfection studies of the immobilized photocatalyst film both in the absence and presence of antibiotic (Initial concentration of *E. coli* = 10^8^ CFU/mL, 1 ppm CIP, under sunlight). **(b)** Kinetics plot of disinfection in the absence of antibiotic and **(c)** Kinetics plot of disinfection in the presence of antibiotic.

Figure 13a shows the disinfection of *E. coli* using the /EPS film under sunlight both in the absence and presence of CIP. In the absence of CIP, 6.357 log reduction (99.9999%), and in the presence of CIP, 7.302 log reduction (99.99999%) was observed at the end of 180 min. These results indicate that the immobilized photocatalytic film is quite effective in disinfecting the target micro-organism as these results are in close proximity to the results obtained in the slurry form (7.046 log reduction). The kinetics were studied using the following equation (Eq. 3)^85^ using GlnaFit tool. From the kinetics plot of {log_10_ (N) (CFU/mL)} vs. time (Fig. 13b,c for disinfection in the absence and presence of CIP respectively), *k*_*ma*x_ and R^2^ values are determined (see Table 7, Supporting Information—Sect. S8) which has a shoulder (initial flat portion), linear (center portion), and tail (final flat portion).

3 $$N\left(t\right)=\left(\frac{\left(N\left(0\right)-{N}_{res}\right)\times \text{exp}\left(-{k}_{max}t\right)\times (\text{exp}({k}_{max}\times Sl))}{(1+(\text{exp}{(k}_{\mathit{max}}\times Sl)-1)\times \text{exp}({-k}_{max }t)))}\right)+ {N}_{res},$$

where *N*(t) = cell concentration at time t (CFU/mL), *N*(0) = initial cell concentration (CFU/mL), t = reaction time (min), *k*_max_ = rate constant (min^−1^), *Sl* = Shoulder length (as calculated by the tool), *N*_res_ = more resistant subpopulation (tail portion, as calculated by the tool).

The *k*_*ma*x_ values are higher for disinfection in the presence of CIP (antibiotic) when compared to disinfection in the absence of CIP. These results suggest that the disinfection ability of the immobilized film is better even in the presence of antibiotic (CIP), which is essential in the real-water matrix application as it contains both antibiotic residues and superbugs.

The real water samples were spiked with the CIP antibiotic and *E. coli* to determine the performance of the B_0.8_Ce_0.2_TiO_2_/EPS film in the real water matrices. The river water was collected from the Kumaradhara River (Subramanya, Karnataka, India).

**Figure 14:**
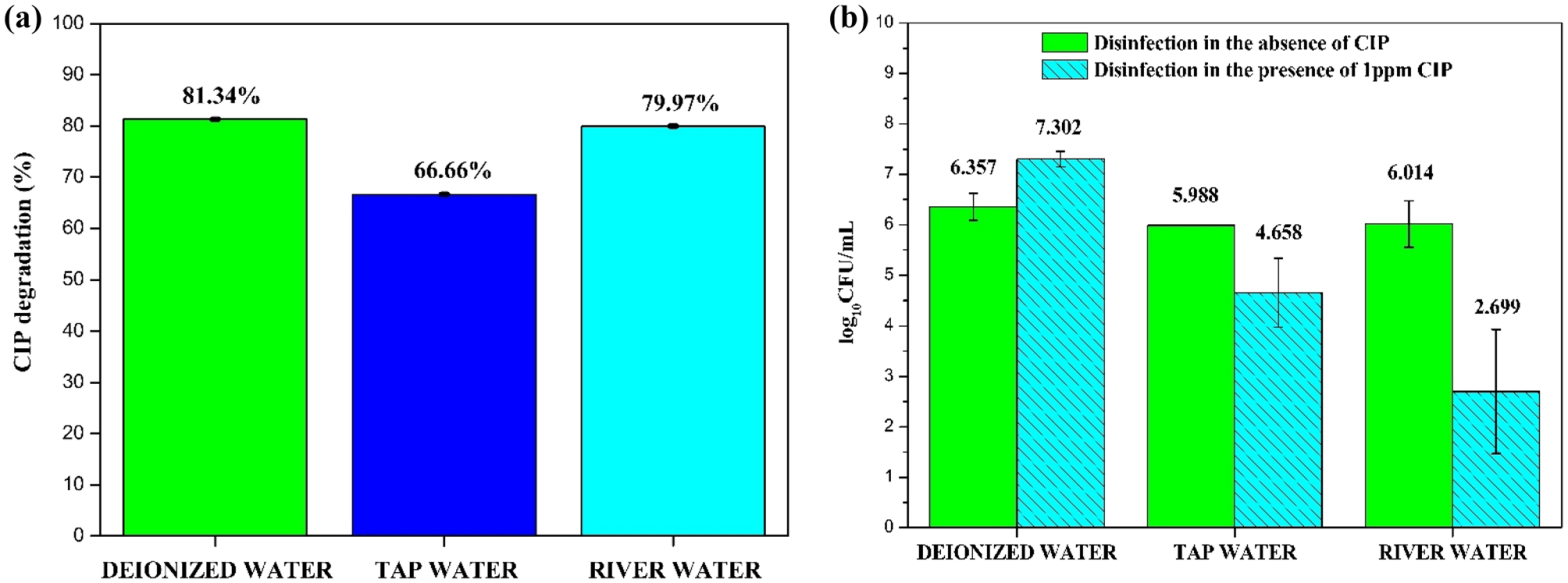
**(a)** CIP degradation using real water samples (10 ppm CIP, 180 min, under sunlight). **(b)** Disinfection in the absence and presence of 1 ppm CIP using real water samples (180 min, under sunlight).

The degradation and disinfection (both in the absence and presence of CIP) performance slightly decreased in the real water samples (Fig. 14a,b) when compared to the deionized water. The presence of anions and humic acid (natural organic matter) might hinder the degradation efficiency in the real water samples. As mentioned in the literature^86^, the anions and natural organic matter might react with the generated reactive species and result in a weaker oxidizing agent. Also, the anions could act as scavengers for ^**⋅**^OH and h^+^ species^87,88^.

### Performance of the photocatalyst in suspended and immobilized forms under sunlight and UV-A irradiation

**Figure 15:**
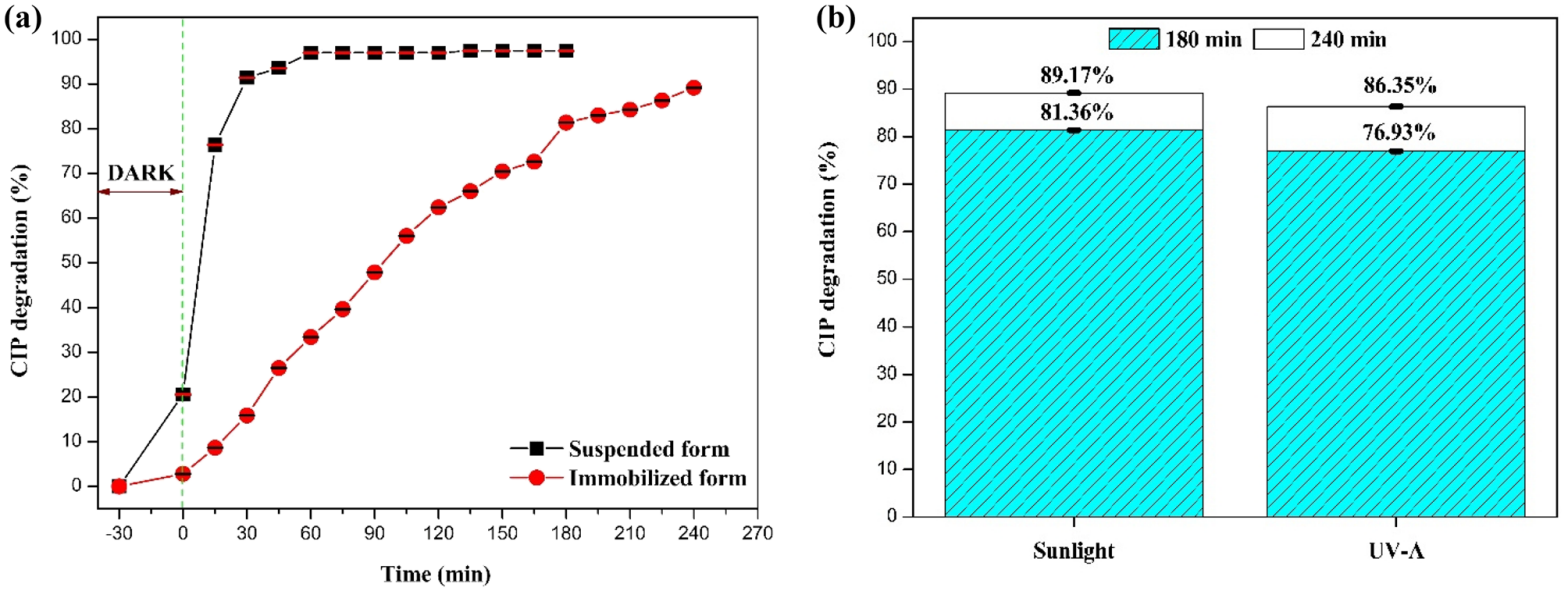
**(a)** Comparison of CIP degradation efficiency in suspended and immobilized forms, **(b)** Comparison of CIP degradation efficiency under sunlight and UV-A irradiation (immobilized form).

It is well-known that the suspended form has better photocatalytic performance than the immobilized form due to the available larger surface area in the suspended form. The degradation efficiency obtained in the immobilized form (89.17%, 240 min) is slightly lesser than that of the suspended form (97.43%, 180 min) (Fig. 15a). An increase in the treatment time/longer timeframe can be considered in order to achieve higher efficiency. Moreover, in large-scale application, the immobilized form helps in the reuse of catalysts, thus reducing operating costs.

From Fig. 15b for immobilized form, the degradation efficiency of CIP under sunlight is found to be slightly higher than that under UV-A irradiation. Similar results are reported in the literature^89^.

### Batch scale-up study, effect of H_2_O_2_ and degradation of other antibiotics

**Figure 16:**
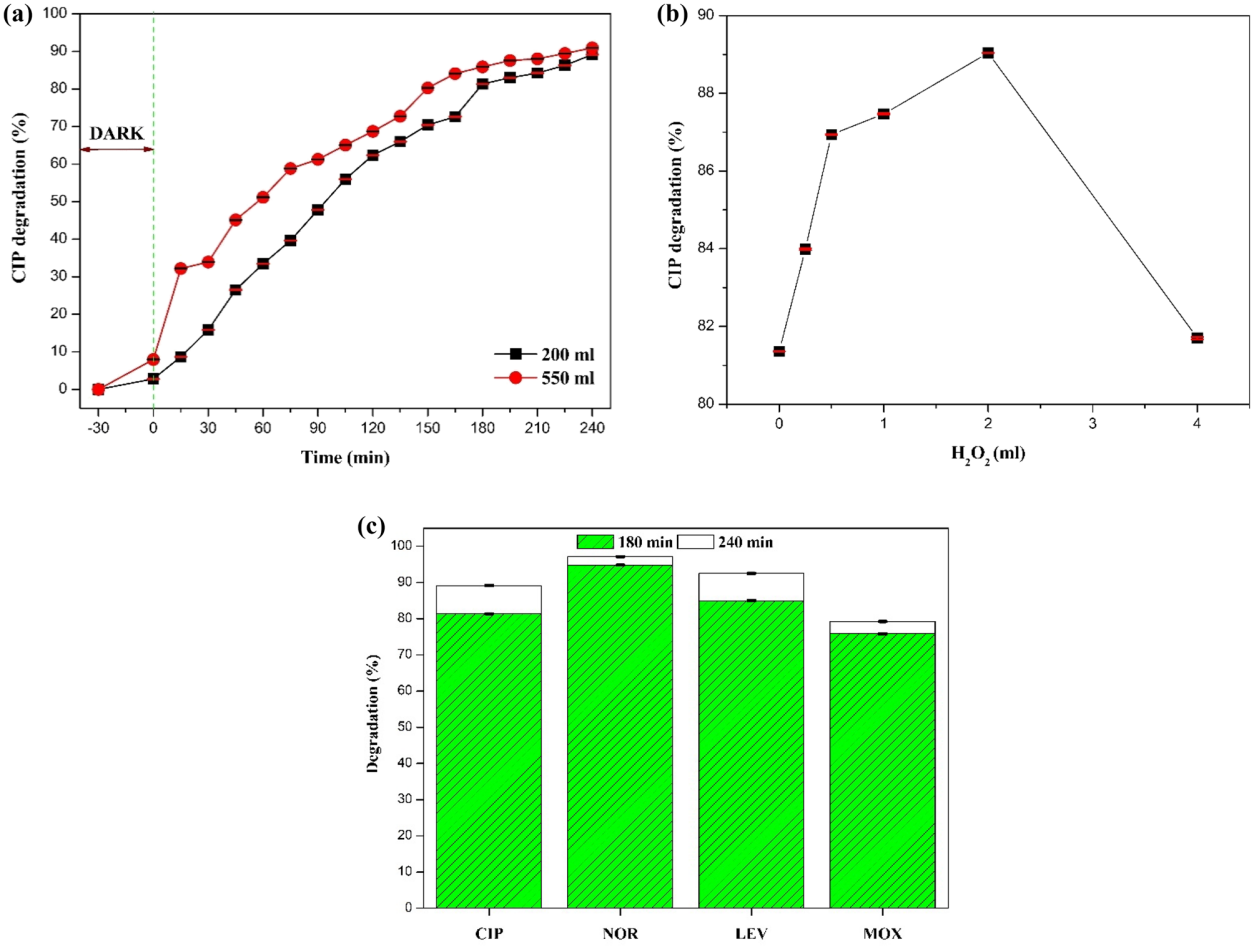
**(a)** Batch reactor scale-up studies using 550 mL of CIP solution under sunlight, **(b)** effect of H_2_O_2_ as an oxidizing agent for 200 ml reactor, and **(c)** degradation of other antibiotics using the B_0.8_Ce_0.2_TiO_2_ immobilized film.

A batch scale-up study was performed 550 mL of the CIP solution to determine the effectiveness of the B_0.8_Ce_0.2_TiO_2_ immobilized film for treating a larger volume of pollutant solution. The scale-up criteria employed is the ratio of the surface area of the polymer film to reactor volume, and the optimized conditions of the 200 mL were used for the 550 mL reactor. The results are shown in Fig. 16a. The degradation efficiency in both reactor volumes is fairly close to each other, thus confirming the scale-up criteria employed here. However, a slightly higher degradation for the larger reactor volume (~ 86% degradation at 180 min compared to 225 min for the smaller reactor volume) was observed.

To enhance the degradation activity hydrogen peroxide (H_2_O_2_, an oxidizing agent) was used, and its effect on CIP degradation (at the optimum condition of 20 wt% of catalyst dosage with 200 mL of 10 ppm CIP, and 180 min under sunlight) was examined by varying the concentrations of H_2_O_2_ as 0.25, 0.5, 1, 2, and 4 mL per 200 mL CIP solution. In the photocatalytic degradation process, H_2_O_2_ has a dual role. It can act as an e^−^ scavenger (accepts e^−^ from the conduction band), and it also forms **·**OH^90,91^. As seen from Fig. 16b, with an increase in the H_2_O_2_ concentrations up to 2 mL, the CIP degradation efficiency increased from 81.36 to 89.04% due to the increase in the production of **·**OH by H_2_O_2_. However, with an increase in the H_2_O_2_ concentration (4 mL), CIP degradation efficiency decreased to 81.07% as scavenging of **·**OH occurs due to the reaction between the excess amount of H_2_O_2_ and **·**OH, according to the following equations (Eqs. 4, 5)^86,92^.

4 $${\text{H}}_{2}{\text{O}}_{2}+{\text{OH}}^{\dot{}}\to {{\text{HO}}_{2}}^{\dot{}}+ {\text{H}}_{2}{\text{O}},$$

5 $${{\text{HO}}_{2}}^{\dot{}}+ {\text{OH}}^{\dot{}} \to {\text{O}}_{2}+ {\text{H}}_{2}{\text{O}}.$$

Figure 16c shows the degradation of other antibiotics, such as norfloxacin (NOR), levofloxacin (LEV), and moxifloxacin (MOX), performed using the B_0.8_Ce_0.2_TiO_2_ immobilized film under sunlight. The degradation efficiency at the end of 240 min is of the following increasing order: NOR (97.13%) > LEV (92.53%) > CIP (89.17%) > MOX (79.26%). These significant degradation results suggest the effectiveness of the B_0.8_Ce_0.2_TiO_2_ immobilized film for the degradation of a broad range of fluoroquinolone antibiotics.

## Conclusions

In this study, a codoped solar-light active photocatalyst (B_0.8_Ce_0.2_TiO_2_) was immobilized using waste EPS beads. This inert support being buoyant allows for effective utilization of sunlight, and upcycling of the waste EPS reduces the risk of white pollution. The B_0.8_Ce_0.2_TiO_2_/EPS film was employed for the degradation of ciprofloxacin (antibiotic) and disinfection of *E. coli*. From contact angle measurements, after loading of B_0.8_Ce_0.2_TiO_2_ photocatalyst, the EPS film behaved as hydrophilic in nature. From XPS analysis, the actual amount of the immobilized photocatalyst was in accordance with the intended/calculated amount during the preparation of the film. The effect of the mixture of solvents in film preparation, the effect of catalyst dosage, and the effect of CIP concentrations on the degradation was studied. The degradation was confirmed through spectrophotometer analysis, TOC reduction, and microbiologically by the residual antimicrobial activity of the degraded ciprofloxacin sample (well diffusion method). A maximum degradation of 89.17% was observed 240 min respectively at an optimum catalyst dosage of 20 wt% of B_0.8_Ce_0.2_TiO_2_. A significant TOC reduction of 84.41% was observed at the end of 240 min. The loss of antimicrobial activity of the degraded CIP sample was confirmed from the decrease of inhibition zones with an increase in the treatment time. From reusability studies, the B_0.8_Ce_0.2_TiO_2_/EPS film was found to be stable even after five times of reuse and its application for large-scale purposes. No morphological, structural, and chemical changes in the film were observed (after five times of reuse) as confirmed from FESEM, XRD, and FTIR analysis, respectively. Minute traces of doped elements (B and Ce) were observed from ICP-OES analysis (leaching study). Effective and promising results were observed for the degradation and disinfection studies carried out using real water samples. A slightly higher degradation (90.96% at the end of 240 min) was observed for the larger reactor volume (550 mL). These immobilized films were effective against a broad range of fluoroquinolone antibiotics. These results suggest the efficacy of the EPS film in terms of both degradation and disinfection under sunlight which can serve as a sustainable tool for the emerging global antimicrobial resistance. Effectiveness of B_0.8_Ce_0.2_TiO_2_/EPS film using real water samples, and broad range of antibiotics clearly indicates its suitability for real-world applications with mixture of antibiotics in waterbodies. In comparison with similar works involving polystyrene or other supports for immobilization (Tables 1, 2, Supporting Information, S1), it is evident that the B_0.8_Ce_0.2_TiO_2_/EPS film is as efficient or perhaps better than some of the latest generation photocatalysts. Moreover, the upcycled EPS beads contributes to minimizing the ‘white pollution’ as mentioned earlier. Further, these immobilized films can be employed for simultaneous degradation and disinfection studies which are essential to solve the problem of antimicrobial resistance. Also, reactor studies can be carried out using the EPS film for large-scale applications.

### Supplementary Information

Supplementary Information.

## Data Availability

All data generated or analysed during this study are included in this published article (and its Supplementary Information files).
