# Supplementary material for: Upcycling of waste EPS beads to immobilized codoped TiO2 photocatalysts for ciprofloxacin degradation and E. coli disinfection under sunlight
Source: Sci Rep. 2023 Sep 5;13:14631. doi: 10.1038/s41598-023-41705-1 (PMC10480149; doi:10.1038/s41598-023-41705-1)
Supplement: Supplementary file 1 — Supplementary Information. [file 41598_2023_41705_MOESM1_ESM.docx]

**S1. Comparison with literature**

Some of the studies reported for degradation of dyes and disinfection using polystyrene and expanded polystyrene (EPS) beads have been summarized below in Table 1. Table 2 summarizes the studies involving dyes, antibiotic and disinfection using other supports for immobilization. Less or no studies have been reported on use of immobilized polystyrene photocatalysts for treatment of antibiotic solutions.

**Table 1.** Photocatalytic studies with polystyrene as a support for immobilization

| **PHOTOCATALYST** | **PREPARATION METHOD** | **LIGHT SOURCE** | **DEGRADATION/**  **DISINFECTION** | **REFERENCE** |
| --- | --- | --- | --- | --- |
| Waste EPS–TiO_2_ composite (W-TiEPS) | Dissolving using acetone and water | UV-A | Methylene blue and  Cr (VI) reduction | 1 |
| Polystyrene beads with TiO_2_ film | Physical vapour deposition | UV-B | Methylene blue  *E.coli*  C_0_ - 6×10^9^ cells/mL  Disinfection - 100%  T - 45 min | 2 |
| N-TiO_2_ /  polystyrene | Sol-gel,  Solvent-casting | Visible light  (8W lamps) | Methylene blue  *E.coli*  C_0_ -10^5^ CFU/mL  Disinfection - 97%  T - 30 min | 3 |
| TiO_2_ /  polystyrene | Solvent-casting | UV-A | Methyl orange and methylene blue | 4 |
| TiO_2_ /  polystyrene | Solvent-casting | UV-C | Remazol Turquoise Blue | 5 |
| TiO_2_ /  polystyrene | Solvent-casting | UV-A | Methylene blue | 6 |
| TiO_2_ P25 grafted on expanded polystyrene (EPS) beads | Grafting | UV-A | Methylene blue, indigo carmine and drimaren red | 7 |
| Ag doped TiO_2_ polystyrene | Liquid impregnation,  Solvent-casting | UV-C,  Sunlight | Methylene blue | 8 |
| ZnO supported on polystyrene pellets | Solvent-casting | UV-LED | Caffeine and paracetamol | 9 |
| TiO_2_-coated polystyrene beads | Sprinkle and heat-fixing | UV-A | Methylene blue | 10 |
| B_0.8_Ce_0.2_TiO_2_ /EPS film | EDTA-citrate method,  Dissolving EPS beads using acetone and ethanol | Sunlight | Ciprofloxacin (CIP)  C_0_ - 10 mg/L  Degradation - 81.36%, 89.17%  TOC reduction - 46.41%, 84.41%  T - 180 min, 240 min  *E.coli*  C_0_ -10^8^ CFU/mL  Disinfection - 99.9999%, 99.99999% (in the presence of 1ppm of CIP)  T - 180 min | This work |

**Table 2.** Photocatalytic studies involving dyes, antibiotics and disinfection using other supports for immobilization

| **PHOTOCATALYST** | **PREPARATION METHOD** | **LIGHT SOURCE** | **EXPERIMENTAL CONDITIONS/**  **MAJOR RESULTS** | **REFERENCE** |
| --- | --- | --- | --- | --- |
| P/Ag/Ag_2_O/Ag_3_PO_4_/ TiO_2_ (PAgT) | Sol-gel/hydrothermal two-step method | Visible light | *E.coli*, *Salmonella*, *S.aureus*, and *Enterococcus*  C_0_ - 10^7^ CFU/mL  Disinfection - 100%  T – 20 min, 30 min, 180 min, and 360 min | 11 |
| TiO_2_ films on stainless steel meshes | Metal-organic chemical vapour deposition | UV-A | Warfarin, Trimethoprim, Carbamazepine, Metoprolol,  and Gemfibrozil | 12 |
| TiO_2_ immobilized onto glass spheres | Dip-coating | Sunlight  [Pilot  compound parabolic collector (CPC) plant] | Acetaminophen, Caffeine, Ofloxacin, Antipyrine, Sulfamethoxazole, Carbamazepine, Flumequine, Keterolac, Atrazine, Isoproturon, Hydroxybiphenyl, Diclofenac, Ibuprofen, Progesterone, Triclosan | 13 |
| TiO_2_ / quartz filter / porous titanium substrate | Sol-gel | UV-LED | Carbamazepine and atorvastatin | 14 |
| N- TiO_2_ immobilized on glass spheres | Using sodium silicate as binder | Visible light  (500 W Xenon lamp) | Ciprofloxacin  C_0_ - 20 mg/L  Degradation - 97.5%  T - 180 min  Analysis - UPLC-Q-TOF MS | 15 |
| Hybrid acrylic TiO_2_ films | Casting TiO_2_ pickering stabilized acrylic latexes | Simulated sunlight | *E.coli*  C_0_ - 10^6^ CFU/mL  Disinfection - 100%  T - 200 min | 16 |
| WS_2_ decorated and immobilized on  chitosan and polycaprolactone | Electrospinning | UV-A | Neomycin | 17 |
| TiO_2_ supported on autoclaved cellular concrete | Oven dried | UV-A | Indigo carmine | 18 |
| TiO_2_-reduced graphene oxide coated side-glowing optical fibers | Polymer assisted hydrothermal deposition | UV-A and visible light | Ibuprofen, Carbamazepine, and Sulfamethoxazole | 19 |
| Graphitic carbon nitride on polyethylene terephthalate nanofibres | Thermal decomposition of urea,  Electrospinning | Sunlight | Sulfaquinoxaline | 20 |
| TiO_2_ immobilized on cylindrical borosilicate glass tubes | Dip coating | Simulated sunlight | Acetaminophen, Diclofenac, Carbamazepine, Atenolol, Propanolol, Albuterol, Ofloxacin, Ciprofloxacin, Azithromycin, Erythromycin, Hydrochlorothiazide, Furosemide | 21 |
| TiO_2_-Calcium alginate beads | Entrapping | UV-A | Chlorhexidine digluconate | 22 |
| Ce-TiO_2_ immobilized on porous glass | Dip coating | Visible light  (300 W Xenon lamp) | Methyl orange and Rhodamine B | 23 |
| TiO_2_ films immobilized on inner surface of borosilicate glass tube of  CPC reactor | Dip coating | UV-A | *E.coli*  C_0_ - 10^6^ CFU/mL  Disinfection - 99.999%  T - 5 h | 24 |
| Polyethylene (PE)-TiO_2_ films | Immersing PE film in TiO_2_ suspension | UV-A | Methylene blue | 25 |
| Glass fiber (GF) TiO_2_-Cu mats | Dipping GF mats in TiO_2_ suspension | UV-C | Methylene blue  *E.coli* K12  C_0_ - 10^6^ CFU/mL  Disinfection - 100%  T - 180 min | 26 |
| Polyethylene (PE)-TiO_2_ films | Direct current magnetron sputtering | UV-C | *E.coli* K12  C_0_ - 10^6^ CFU/mL  Disinfection - 100%  T - 180 min | 27 |
| CuO_x_-TiO_2_-PET | Direct current magnetron sputtering | Simulated sunlight | *E.coli*  C_0_ - 4 ×10^6^ CFU/mL  Disinfection - 100%  T - 150 min | 28 |
| TiO_2_-PANI/Cork | Using binder and cork | Sunlight | Methyl orange | 29 |
| TiO_2_/PMMA (poly methyl methacrylate) film | Inverted phase | UV-A | Methylene blue | 30 |
| TiO_2_/Calcium alginate beads | Dripping (Ionotropic gelation) | UV-A | Tartrazine | 31 |

C_0_ – Initial concentration; T- treatment time

**S2. Floating photocatalyst and synthesis of codoped B_0.8_Ce_0.2_TiO_2_ photocatalyst**

**Figure S1.** B_0.8_Ce_0.2_TiO_2_ /EPS film (floating photocatalyst – which comes in contact with air-liquid interface)

The B_0.8_Ce_0.2_TiO_2_ catalyst was synthesized using the facile green EDTA-citrate sol-gel method which was earlier identified as the best performing codoped photocatalyst in our laboratory 32 and the synthesis steps are as follows to synthesize 10 g of catalyst.

EDTA (24.15% w/v) is dissolved in water using ammonia + cerium nitrate hexahydrate (0.12% w/v) and boric acid (0.21% w/v) precursor solutions + TiO_2_ solution (19.66% w/v)

Citric acid (‘x’ g - as calculated based on the following mole ratio) added to above solution (kept under constant stirring), pH adjusted to 9.0 using ammonia

Mole ratio of 1:1:1.5 (metal/boric acid: EDTA: citric acid) was maintained

Mixture - heated and stirred continuously until the formation of an organometallic gel

Gel - dried at 150°C for 24 h in a hot air oven

Dried powder - subjected to a two-step calcination process; 350°C for 12 h followed by 600°C for 5 h to remove all organic impurities and stored for future use

**Scheme 1.** Synthesis of codoped photocatalyst (B_0.8_Ce_0.2_TiO_2_

**S3. Characterization of catalyst and XPS analysis composition table**

**Table 3.** Results of catalyst characterization as obtained from DLS, DRS and BET Surface area analyses published in [32]

| **Catalyst** | **Particle Size (nm)** | **Band Gap Energy (eV)** | **BET Surface area (m^2^/g)** |
| --- | --- | --- | --- |
| B_0.8_Ce_0.2_TiO_2_ | **15.2** | **2.63** | **32.1** |

**Table 4.** Elemental composition of B_0.8_Ce_0.2_TiO_2_ immobilized photocatalyst

| **Immobilized photocatalyst (B_0.8_Ce_0.2_TiO_2_)** | **Ti** | **O** | **B** | **Ce** |
| --- | --- | --- | --- | --- |
| **At. (%)** | 29.28 | 69.79 | 0.74 | 0.19 |
| **Wt. (%)** | 67.01 | 32 | 0.23 | 0.76 |
| **Wt. (g)** | 0.402 | 0.192 | 0.00138 | 0.00456 |

Total Wt. (g) = 0.402 + 0.192 + 0.00138 + 0.00456 = 0.599 g

$Wt. \left( g \right)=Total Wt. (g)\times\frac{Wt. (\%)}{100}$

(20 wt.% optimum loading) [$0.6 g of catalyst=3\times\frac{20}{100}$]

$$0.599=3\times\frac{Wt. (\%)}{100}$$

Wt. (%) = 19.96 %

**S4. Kinetics of CIP degradation**

**Table 5.** Kinetics data of CIP degradation

| **Immobilized photocatalyst** | ***k* × 10^−3^ (min^−1^)** | | | | **R^2^** | | | |
| --- | --- | --- | --- | --- | --- | --- | --- | --- |
|  | **10 ppm** | **20 ppm** | **30 ppm** | **40 ppm** | **10 ppm** | **20 ppm** | **30 ppm** | **40 ppm** |
| B_0.8_Ce_0.2_TiO_2_ | 8.13 | 5.58 | 2.00 | 1.62 | 0.9906 | 0.9902 | 0.9837 | 0.9630 |

**S5. Reusability studies**


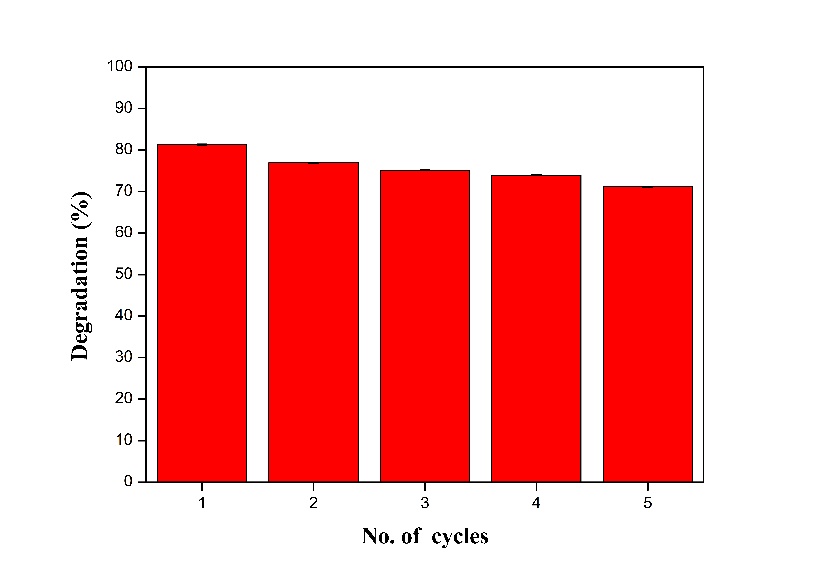


**Figure S2.** Reusability of EPS film performed for five consecutive cycles

**S6. Antimicrobial activity of the degraded CIP sample**

Qualitative assay to determine the antimicrobial activity of the degraded CIP sample was performed using *E.coli* (MTCC 9541) as test organism by the well diffusion method. 100 μL of bacterial suspension was spread onto the agar plate and the wells were loaded with 100 μL of the degraded CIP samples collected at various time intervals. Sterile distilled water was used as the control. The plates were then incubated for 24 h at 37℃. Inhibition zones were observed up to 180 min and after which no inhibition zones were observed (Figure S3).


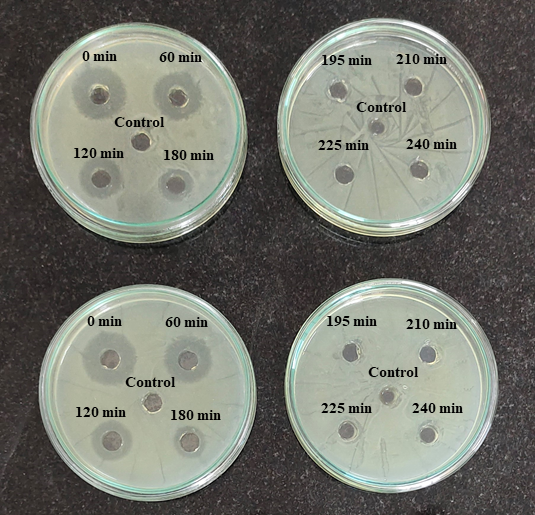


**Figure S3.** Antimicrobial activity of the degraded CIP sample

**S7. Degradation products**

The m/z values of CIP degraded products obtained from LC-MS analysis were compared with that of the literature products and is given in the table 6 below.

**Table 6.** Degradation products of CIP

| Degradation Products | | Experimental m/z | Literature m/z | | Structure | References |
| --- | --- | --- | --- | --- | --- | --- |
| CIP | 331 | | | 331 | **Piperazine moiety**  **Quinoline moiety**  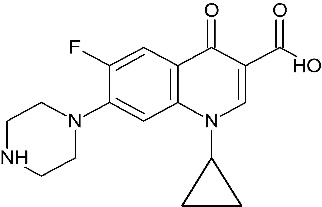 | 33–38 |
| D1 | 338 | | | 334 | 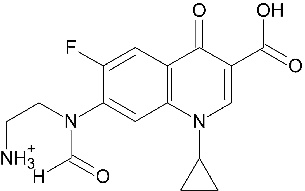 | 36,37,39 |
| D2 | 314 | | | 315 | 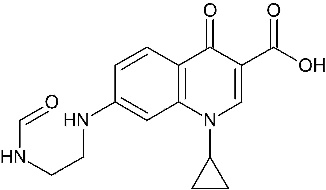 | 36 |
| D3 | 292 | | | 291 | 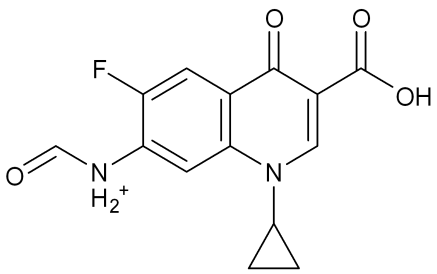 | 37 |
| D4 | 282 | | | 283 | 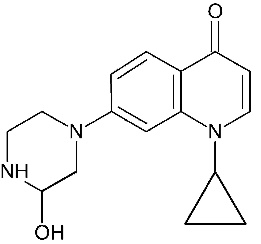 | 35 |
| D5 | 262 | | | 263 | 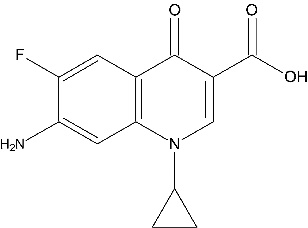 | 33,36,37,39,40 |
| D6 | 251 | | | 251 | 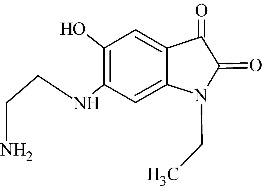 | 41 |
| D7 | 227 | | | 229 | 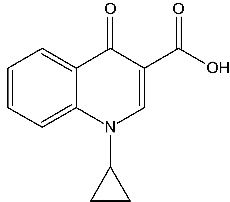 | 35 |
| D8 | 201 | | | 200 | 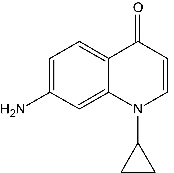 | 35 |
| D9 | 59 | | | 59 | 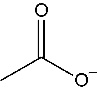 | 42 |

**S8. Disinfection kinetics**

**Table 7.** Kinetics of *E.coli* disinfection in the absence and presence of CIP using B_0.8_Ce_0.2_TiO_2_ catalyst

| PHOTOCATALYSTS | *k_max_* (min^−1^) | | R^2^ | |
| --- | --- | --- | --- | --- |
|  | **Absence of CIP** | **Presence of 1ppm CIP** | **Absence of CIP** | **Presence of 1 ppm CIP** |
| B_0.8_Ce_0.2_TiO_2_ immobilized film | 0.14 | 0.21 | 0.9824 | 0.9983 |

**References**

(1) Lee, Y. J.; Lee, C. G.; Kang, J. K.; Park, S. J.; Alvarez, P. J. J. Simple Preparation Method for Styrofoam-TiO_2_ Composites and Their Photocatalytic Application for Dye Oxidation and Cr (VI) Reduction in Industrial Wastewater. *Environ. Sci. Water Res. Technol.* **2021**, *7* (1), 222–230. https://doi.org/10.1039/d0ew00787k.

(2) Varnagiris, S.; Urbonavicius, M.; Sakalauskaite, S.; Daugelavicius, R.; Pranevicius, L.; Lelis, M.; Milcius, D. Floating TiO_2_ Photocatalyst for Efficient Inactivation of *E. Coli* and Decomposition of Methylene Blue Solution. *Sci. Total Environ.* **2020**, *720*. https://doi.org/10.1016/j.scitotenv.2020.137600.

(3) Ata, R.; Sacco, O.; Vaiano, V.; Rizzo, L.; Tore, G. Y.; Sannino, D. Visible Light Active N-Doped TiO_2_ Immobilized on Polystyrene as Efficient System for Wastewater Treatment. *J. Photochem. Photobiol. A Chem.* **2017**, *348*, 255–262. https://doi.org/10.1016/j.jphotochem.2017.08.054.

(4) Singh, S.; Chaki, A.; Chand, D. P.; Raghuwanshi, A.; Singh, P. K. A Novel Polystyrene-Supported Titanium Dioxide Photocatalyst for Degradation of Methyl Orange and Methylene Blue Dyes under UV Irradiation. *J. Chem. Eng.* **2013**, *28* (1), 9–13. <https://doi.org/10.3329/jce.v28i1.18103>.

(5) Das, S.; Mahalingam, H. Reusable Floating Polymer Nanocomposite Photocatalyst for the Efficient Treatment of Dye Wastewaters under Scaled-up Conditions in Batch and Recirculation Modes. *J. Chem. Technol. Biotechnol.* **2019**, *94* (8), 2597–2608. https://doi.org/10.1002/jctb.6069.

(6) Singh, S.; Singh, P. K.; Mahalingam, H. An Effective and Low-Cost TiO_2_ / Polystyrene Floating Photocatalyst for Environmental Remediation. *Int. J. Environ. Res.* **2015**, *9* (2), 535–544. https://doi.org/10.22059/IJER.2015.927.

(7) Magalhães, F.; Lago, R. M. Floating Photocatalysts Based on TiO_2_ Grafted on Expanded Polystyrene Beads for the Solar Degradation of Dyes. *Sol. Energy* **2009**, *83* (9), 1521–1526. https://doi.org/10.1016/j.solener.2009.04.005.

(8) Singh, S.; Singh, P. K.; Mahalingam, H. Novel Floating Ag ^+^ ‑ Doped TiO_2_ / Polystyrene Photocatalysts for the Treatment of Dye Wastewater. **2014**. *53* (42), 16332–16340. <https://doi.org/10.1021/ie502911a>.

(9) Vaiano, V.; Matarangolo, M.; Sacco, O. UV-LEDs Floating-Bed Photoreactor for the Removal of Caffeine and Paracetamol Using ZnO Supported on Polystyrene Pellets. *Chem. Eng. J.* **2018**, *350* (May), 703–713. https://doi.org/10.1016/j.cej.2018.06.011.

(10) Fabiyi, M. E.; Skelton, R. L. Photocatalytic Mineralisation of Methylene Blue Using Buoyant TiO_2_ -Coated Polystyrene Beads. *J. Photochem. Photobiol. A Chem.* **2000**, *132*, 121–128. https://doi.org/10.1016/s1010-6030(99)00250-6.

(11) Liu, N.; Ming, J.; Sharma, A.; Sun, X.; Kawazoe, N.; Chen, G.; Yang, Y. Sustainable Photocatalytic Disinfection of Four Representative Pathogenic Bacteria Isolated from Real Water Environment by Immobilized TiO_2_-Based Composite and Its Mechanism. *Chem. Eng. J.* **2021**, *426* (June), 131217. https://doi.org/10.1016/j.cej.2021.131217.

(12) Murgolo, S.; Yargeau, V.; Gerbasi, R.; Visentin, F.; El Habra, N.; Ricco, G.; Lacchetti, I.; Carere, M.; Curri, M. L.; Mascolo, G. A New Supported TiO_2_ Film Deposited on Stainless Steel for the Photocatalytic Degradation of Contaminants of Emerging Concern. *Chem. Eng. J.* **2017**, *318*, 103–111. https://doi.org/10.1016/j.cej.2016.05.125.

(13) Miranda-García, N.; Maldonado, M. I.; Coronado, J. M.; Malato, S. Degradation Study of 15 Emerging Contaminants at Low Concentration by Immobilized TiO_2_ in a Pilot Plant. *Catal. Today* **2010**, *151* (1–2), 107–113. https://doi.org/10.1016/j.cattod.2010.02.044.

(14) Arlos, M. J.; Hatat-Fraile, M. M.; Liang, R.; Bragg, L. M.; Zhou, N. Y.; Andrews, S. A.; Servos, M. R. Photocatalytic Decomposition of Organic Micropollutants Using Immobilized TiO_2_ having Different Isoelectric Points. *Water Res.* **2016**, *101*, 351–361. https://doi.org/10.1016/j.watres.2016.05.073.

(15) Xing, X.; Du, Z.; Zhuang, J.; Wang, D. Removal of Ciprofloxacin from Water by Nitrogen Doped TiO_2_ Immobilized on Glass Spheres: Rapid Screening of Degradation Products. *J. Photochem. Photobiol. A Chem.* **2018**, *359*, 23–32. https://doi.org/10.1016/j.jphotochem.2018.03.026.

(16) Bonnefond, A.; González, E.; Asua, J. M.; Leiza, J. R.; Kiwi, J.; Pulgarin, C.; Rtimi, S. New Evidence for Hybrid Acrylic/TiO_2_ Films Inducing Bacterial Inactivation under Low Intensity Simulated Sunlight. *Colloids Surfaces B Biointerfaces* **2015**, *135*, 1–7. https://doi.org/10.1016/j.colsurfb.2015.07.034.

(17) Fakhri, A.; Gupta, V. K.; Rabizadeh, H.; Agarwal, S.; Sadeghi, N.; Tahami, S. Preparation and Characterization of WS_2_ Decorated and Immobilized on Chitosan and Polycaprolactone as Biodegradable Polymers Nanofibers: Photocatalysis Study and Antibiotic-Conjugated for Antibacterial Evaluation. *Int. J. Biol. Macromol.* **2018**, *120*, 1789–1793. https://doi.org/10.1016/j.ijbiomac.2018.09.207.

(18) Andrade, F. V. De; de Lima, G. M.; Augusti, R.; da Silva, J. C. C.; Coelho, M. G.; Paniago, R.; Machado, I. R. A Novel TiO_2_/Autoclaved Cellular Concrete Composite: From a Precast Building Material to a New Floating Photocatalyst for Degradation of Organic Water Contaminants. *J. Water Process Eng.* **2015**, *7*, 27–35. https://doi.org/10.1016/j.jwpe.2015.04.005.

(19) Lin, L.; Wang, H.; Xu, P. Immobilized TiO2-Reduced Graphene Oxide Nanocomposites on Optical Fibers as High Performance Photocatalysts for Degradation of Pharmaceuticals. *Chem. Eng. J.* **2017**, *310*, 389–398. https://doi.org/10.1016/j.cej.2016.04.024.

(20) Qin, D.; Lu, W.; Wang, X.; Li, N.; Chen, X.; Zhu, Z.; Chen, W. Graphitic Carbon Nitride from Burial to Re-Emergence on Polyethylene Terephthalate Nanofibers as an Easily Recycled Photocatalyst for Degrading Antibiotics under Solar Irradiation. *ACS Appl. Mater. Interfaces* **2016**, *8* (39), 25962–25970. https://doi.org/10.1021/acsami.6b07680.

(21) Palacios-Villarreal, C.; Manzano, M.; Jos, J.; Blanco, E.; Ramírez, M.; Levchuk, I. Photocatalytic Degradation of Pharmaceutically Active Compounds (PhACs) in Urban Wastewater Treatment Plants Effluents under Controlled and Natural Solar Irradiation Using Immobilized TiO_2_. *Sol. Energy* **2020**, *208* (March), 480–492. https://doi.org/10.1016/j.solener.2020.08.028.

(22) Sarkar, S.; Chakraborty, S.; Bhattacharjee, C. Photocatalytic Degradation of Pharmaceutical Wastes by Alginate Supported TiO_2_ Nanoparticles in Packed Bed Photo Reactor (PBPR ). *Ecotoxicol. Environ. Saf.* **2015**, 1–8. https://doi.org/10.1016/j.ecoenv.2015.02.035.

(23) Shen, C.; Pang, K.; Du, L.; Luo, G. Green Synthesis and Enhanced Photocatalytic Activity of Ce-Doped TiO_2_ Nanoparticles Supported on Porous Glass. **2017**, 1–7. https://doi.org/10.1016/j.partic.2017.01.007.

(24) Alrousan, D. M. A.; Polo-López, M. I.; Dunlop, P. S. M.; Fernández-Ibáñez, P.; Byrne, J. A. Solar Photocatalytic Disinfection of Water with Immobilised Titanium Dioxide in Re-Circulating Flow CPC Reactors. *Appl. Catal. B Environ.* **2012**, *128*, 126–134. https://doi.org/10.1016/j.apcatb.2012.07.038.

(25) Suárez, L.; Pulgarin, C.; Roussel, C.; Kiwi, J. Preparation , Kinetics , Mechanism and Properties of Semi-Transparent Photocatalytic Stable Films Active in Dye Degradation. *"Applied Catal. A, Gen.* **2016**, *516*, 70–80. https://doi.org/10.1016/j.apcata.2016.01.041.

(26) Suárez, L.; Pulgarin, C.; Giannakis, S.; Bensimon, M.; Kiwi, J. New Evidence for Disinfection, Self-Cleaning and Pollutant Degradation Mediated by GF-TiO_2_-Cu Mats under Solar/Visible Light in Mild Oxidative Conditions. *J. Photochem. Photobiol. A Chem.* **2017**, *346*, 351–363. https://doi.org/10.1016/j.jphotochem.2017.06.025.

(27) Rtimi, S.; Sanjines, R.; Andrzejczuk, M.; Pulgarin, C.; Kulik, A.; Kiwi, J. Innovative Transparent Non-Scattering TiO_2_ bactericide Thin Films Inducing Increased *E. Coli* Cell Wall Fluidity. *Surf. Coatings Technol.* **2014**, *254* (July), 333–343. https://doi.org/10.1016/j.surfcoat.2014.06.035.

(28) Rtimi, S.; Pulgarin, C.; Robyr, M.; Aybush, A.; Shelaev, I.; Gostev, F.; Nadtochenko, V.; Kiwi, J. Insight into the Catalyst/Photocatalyst Microstructure Presenting the Same Composition but Leading to a Variance in Bacterial Reduction under Indoor Visible Light. *Appl. Catal. B Environ.* **2017**, *208*, 135–147. https://doi.org/10.1016/j.apcatb.2017.02.043.

(29) Sboui, M.; Nsib, M. F.; Rayes, A.; Swaminathan, M.; Houas, A. TiO_2_–PANI/Cork Composite: A New Floating Photocatalyst for the Treatment of Organic Pollutants under Sunlight Irradiation. *J. Environ. Sci.* **2017**, *60*, 3–13. https://doi.org/10.1016/j.jes.2016.11.024.

(30) Ounas, O.; El Foulani, A. A.; Lekhlif, B.; Jamal-Eddine, J. Immobilization of TiO_2_ into a Poly Methyl Methacrylate (PMMA) as Hybrid Film for Photocatalytic Degradation of Methylene Blue. *Mater. Today Proc.* **2020**, *22*, 35–40. https://doi.org/10.1016/j.matpr.2019.08.068.

(31) Dalponte, I.; de Sousa, B. C.; Mathias, A. L.; Jorge, R. M. M. Formulation and Optimization of a Novel TiO_2_/Calcium Alginate Floating Photocatalyst. *Int. J. Biol. Macromol.* **2019**, *137*, 992–1001. https://doi.org/10.1016/j.ijbiomac.2019.07.020.

(32) Manjunatha, M.; Chandewar, P. R.; Mahalingam, H. Exploring the Synergy of B, Ce Dopants in Codoped Titanium Dioxide Multifunctional Photocatalysts for Antibiotic Degradation and Microbial Disinfection Under Solar Light. *Phys. Status Solidi Appl. Mater. Sci.* **2022**, *219*, 2100581 (1-16). https://doi.org/10.1002/pssa.202100581.

(33) Gupta, A.; Garg, A. Degradation of Ciprofloxacin Using Fenton’s Oxidation: Effect of Operating Parameters, Identification of Oxidized by-Products and Toxicity Assessment. *Chemosphere* **2018**, *193* (November), 1181–1188. https://doi.org/10.1016/j.chemosphere.2017.11.046.

(34) Hubicka, U.; Zmudzki, P.; Talik, P.; Zuromska-Witek, B.; Krzek, J. Photodegradation Assessment of Ciprofloxacin, Moxifloxacin, Norfloxacin and Ofloxacin in the Presence of Excipients from Tablets by UPLC-MS/MS and DSC. *Chem. Cent. J.* **2013**, *7* (1), 1–12. https://doi.org/10.1186/1752-153X-7-133.

(35) Haddad, T.; Kümmerer, K. Characterization of Photo-Transformation Products of the Antibiotic Drug Ciprofloxacin with Liquid Chromatography-Tandem Mass Spectrometry in Combination with Accurate Mass Determination Using an LTQ-Orbitrap. *Chemosphere* **2014**, *115* (1), 40–46. https://doi.org/10.1016/j.chemosphere.2014.02.013.

(36) Maia, A. S.; Ribeiro, A. R.; Amorim, C. L.; Barreiro, J. C.; Cass, Q. B.; Castro, P. M. L.; Tiritan, M. E. Degradation of Fluoroquinolone Antibiotics and Identification of Metabolites/Transformation Products by Liquid Chromatography-Tandem Mass Spectrometry. *J. Chromatogr. A* **2014**, *1333*, 87–98. https://doi.org/10.1016/j.chroma.2014.01.069.

(37) Zhang, X. X.; Li, R.; Jia, M.; Wang, S.; Huang, Y.; Chen, C. Degradation of Ciprofloxacin in Aqueous Bismuth Oxybromide (BiOBr) Suspensions under Visible Light Irradiation: A Direct Hole Oxidation Pathway. *Chem. Eng. J.* **2015**, *274*, 290–297. https://doi.org/10.1016/j.cej.2015.03.077.

(38) Shetty, R.; Chavan, V. B.; Kulkarni, P. S.; Kulkarni, B. D.; Kamble, S. P. Photocatalytic Degradation of Pharmaceuticals Pollutants Using N-Doped TiO_2_ Photocatalyst: Identification of CFX Degradation Intermediates. *Indian Chem. Eng.* **2017**, *59* (3), 177–199. https://doi.org/10.1080/00194506.2016.1150794.

(39) Wang, A.; Wang, H.; Deng, H.; Wang, S.; Shi, W.; Yi, Z.; Qiu, R.; Yan, K. Controllable Synthesis of Mesoporous Manganese Oxide Microsphere Efficient for Photo-Fenton-like Removal of Fluoroquinolone Antibiotics. *Appl. Catal. B Environ.* **2019**, *248* (July 2018), 298–308. https://doi.org/10.1016/j.apcatb.2019.02.034.

(40) An, T.; Yang, H.; Li, G.; Song, W.; Cooper, W. J.; Nie, X. Kinetics and Mechanism of Advanced Oxidation Processes (AOPs) in Degradation of Ciprofloxacin in Water. *Appl. Catal. B Environ.* **2010**, *94* (3–4), 288–294. https://doi.org/10.1016/j.apcatb.2009.12.002.

(41) Shah, N. S.; Khan, J. A.; Sayed, M.; Khan, Z. U. H.; Rizwan, A. D.; Muhammad, N.; Boczkaj, G.; Murtaza, B.; Imran, M.; Khan, H. M.; Zaman, G. Solar Light Driven Degradation of Norfloxacin Using As-Synthesized Bi^3+^ and Fe^2+^ Co-Doped ZnO with the Addition of HSO_5_^−^: Toxicities and Degradation Pathways Investigation. *Chem. Eng. J.* **2018**, *351* (March), 841–855. https://doi.org/10.1016/j.cej.2018.06.111.

(42) Shah, N. S.; Ditta, A.; Ali, J.; Sayed, M.; Ul, Z.; Khan, H.; Murtaza, B.; Iqbal, J.; Ud, S.; Imran, M.; Nadeem, M.; Al-muhtaseb, A. H.; Muhammad, N.; Khan, H. M.; Ghauri, M.; Zaman, G. Toxicities, Kinetics and Degradation Pathways Investigation of Ciprofloxacin Degradation Using Iron-Mediated H_2_O_2_ Based Advanced Oxidation Processes. *Process Saf. Environ. Prot.* **2018**, *117*, 473–482. https://doi.org/10.1016/j.psep.2018.05.020.
